# Diurnal temperature variation in surface soils: an underappreciated control on microbial processes

**DOI:** 10.3389/fmicb.2024.1423984

**Published:** 2024-12-18

**Authors:** Robert A. Sanford, Joanne C. Chee-Sanford, Wendy H. Yang

**Affiliations:** ^1^Department of Earth Science and Environmental Change, University of Illinois at Urbana-Champaign, Urbana, IL, United States; ^2^USDA-ARS, Urbana, IL, United States; ^3^Department of Plant Biology, University of Illinois at Urbana-Champaign, Urbana, IL, United States

**Keywords:** surface soil, diurnal temperature, C-mineralization, reaction rates, microbial adaptation

## Abstract

Large diurnal temperature changes (ΔT) (or the diurnal temperature range (DTR)) in surface soils, ranging from 5°C to often greater than 20°C, are generally acknowledged to occur yet largely disregarded in studies that seek to understand how temperature affects microbially-mediated carbon and nitrogen cycling processes. The soil DTR is globally significant at depths of 30 cm or less, occurring from spring through summer in temperate biomes, during summer periods in the arctic, and year-round in the tropics. Thus, although temperature has long been considered an important factor in controlling microbial processes, our understanding of its effects remains incomplete when considering natural soil temperature cycles. Here we show: (1) documented impacts of diurnal temperature changes on microbial respiration rates; (2) documented observations of surface soils with large DTR (>5°C) that affect soil microbial mineralization rates and redox potentials of important biogeochemical reactions; and (3) direct evidence that the constant temperature regime typically used in laboratory soil incubation studies may therefore lead to mischaracterization of *in situ* temperature controls on microbially influenced processes in the environment. The overall effect is that the DTR yields process rates that are often higher than what has been observed under experimental mean temperature incubation. We suggest that overlooked genetic mechanisms, such as the presence of a circadian clock or thermophilic activity during summer months, are likely contributing to the observed effects of the DTR. To improve our understanding of climate change effects on soil greenhouse gas emissions, nutrient cycling, and other biogeochemical soil processes, we propose a paradigm shift in approach to temperature-inclusive process modeling and laboratory incubation studies that accounts for the important role of natural diurnal temperature fluctuations.

## Introduction

Soils remain a major challenge for microbial process studies due to the highly complex physicochemical characteristics associated with the heterogeneous nature of these environments. In particular, the temperature (T) response of soil microorganisms has become an area of intense investigation in connection with rising global temperature increases due to changing climate (van Gestel et al., 2013; Zhang et al., 2015; AAM, 2017; Li et al., 2017; Hu et al., 2019; Buckeridge et al., 2020; Yin et al., 2020). Despite the general acknowledgment about the cardinal importance of temperature on biogeochemical processes, there yet lacks a consensus on the relationship of microbial growth and activity in response to temperature in soil (Bååth, 2018).

Given the prevailing view from a large body of historical soil science research about the intrinsic nature of soil diurnal temperature patterns, especially in surface soils, there surprisingly exists an anchoring bias where soil microbial studies are largely conducted and interpreted in ways that preclude consideration of natural soil diurnal temperature cycles. Consequently, there remains a perseverance of theory rooted in enzyme kinetics and Arrhenius profiles that continues to propagate seemingly paradoxical interpretations, true even in the relatively few contemporary studies that attempt to address microbial activity in response to temperature variation. Certainly, inadequate experimental approaches are further made insufficient in largely ignoring consideration of fundamental aspects of biology such as circadian rhythms, genetic responses to T variation, and burgeoning knowledge of soil microbial diversity.

### Ideologies concerning temperature responses in soil ecosystems

Here, we present a summary list of familiar assumptions (shown in italics below) of the relationship between T and biogeochemical processes in soil gleaned from research collectives and will subsequently address each with a further compendium of findings that contrast with prevailing thoughts. We note some of these are not explicitly stated as such interpretations, but were expounded to further shed light on the basis for the progression of how temperature response studies have been directed:

1. *Soil microorganisms (Bacteria, Archaea and Fungi) are modeled to respond to temperature as if they were single enzymes, with a predictable activity increase in response to a rise in temperature noting a specific optimum T for the reaction*. Here, the counterpoint is presented from the results of several previous studies with both Fungi and Bacteria that show respiration rates vary in response to diurnal T variation such that the cumulative flux is different than what occurs at a corresponding mean T. A few of the biological factors that are potentially responsible for these observations include circadian clocks tied to diurnal T change rather than light, direct genetic control tied to T variation, and promotion of a diverse microbial community that responds optimally at different points during a diurnal cycle.
2. *The significance of temperature variation in soils is primarily seasonal, and with the exception of early spring freeze–thaw cycles, daily diurnal T variation has little consequence. As such, studies can rely entirely on the seasonal mean T for interpreting T effects on biogeochemical process rates in soil. Consequently, experimental approaches used to evaluate impacts of T increases due to climate change often apply fixed T for incubation experiments.* As a counterpoint, several decades of soil laboratory experiments fundamentally showed fluxes of CO_2_, N_2_O and CH_4_ varied in response to diurnal T changes compared to constant temperature.
3. *Diurnal T change of significant magnitude in surface soils only occurs in relatively few ecosystems on Earth and therefore exert little biological impact. Any process rate variation observed can easily be explained by current enzyme-T models.* Based on this premise, the gradient of diurnal temperature range (DTR) change with soil depth and asymmetry of time above and below the mean T has also been ignored. We will cite references going back over 50 years that report significant diurnal temperature ranges (DTR) of >20°C in surface soils, with maximum temperatures often exceeding 50°C. Notably, these types of observations have been made at all latitudes between the Arctic and Antarctic circles. More recent references bolster the DTR observations through the use of satellite imagery and remote sensing. In addition to showing information from the National Ecological Observatory Network (NEON) sites of DTR in soil along a depth profile, we include temperature data from two Illinois field sites. Similar to the NEON sites, the DTR at depths >26 cm have a small DTR (near the mean T) in comparison to samples taken from 0 to 5 cm where a large DTR up to 15°C is seasonally (e.g., April – October) observed.
4. *Microbial community composition* var*ies with depth in soil and is primarily due to the nature of the carbon pool rather than attributed to any direct effects from T.* Although changes in microbial community composition with soil depth have been noted in previous studies, none have proposed DTR to be a factor and most do not even mention it. We acknowledge that carbon pool variation with depth would still impact the microbial community composition, however we propose that the DTR might even have a larger influence. In partial support of this, we will provide observational data (see Supplementary material) from a four-year study using two agricultural field sites that shows the microbial community structure varies with soil depth but are largely maintained despite variation in season, soil chemical inputs, and plant community. We found the bacterial community present in 0–5 cm soil depth was always significantly different from the bacterial community found at 20–30 cm depth. Among a list of eleven temperature and chemical variables tested, DTR was always included among the highest correlated environmental variables to the patterns of community structure present at both field sites, suggesting its explanatory role in driving microbial community composition.
5. *Studies that recognize diurnal temperature change does occur in soil imply that any biological effects can be understood by simply* var*ying the temperature between the maximum T and the minimum T. For example, a good proxy of diurnal T variation would be to incubate 12 h each at the minimum and maximum T.* We will highlight a number of diurnal T experiments conducted previously that do not follow the natural dynamics of change in T that occurs in surface soils. In addition, the magnitude of the DTR decreases with soil depth, as well the asymmetry of the maximum and minimum T above and below the mean T, respectively. Our observations suggest that the DTR in the top 5 cm reflects the most important region in surface soils and that the best experimental soil incubations should follow the daily T at this depth closely.

In the following sections, we further detail information aimed at challenging these assumptions that appear to prevail in contemporary microbial temperature response studies.

### Microbial metabolic rates are influenced by diurnal T change

The important premise presented here is that soil surface diurnal temperature change creates a cycle legacy that in large part drives the extant community structure that microbes within these communities have surely adapted. Further, it is apparent that the mechanisms of temperature response in microorganisms is not yet comprehensively understood. Paramount evidence indicating temperature responses are present to some extent in the literature but lacked adequate interpretation to extend current hypotheses or simply not referenced accordingly in the context of explaining biological temperature responses in soil. Table 1 summarizes studies conducted using pure cultures that have reported metabolic shifts corresponding to diurnal T in contrast to constant mean T incubations. Note these observations contrast with the current expectation that there should be no effect of an experimentally set DTR verses mean T incubation. Several studies conducted more than 50 years ago established that different fungal species would respond with greater growth rates when incubated under diurnal temperature variation conditions compared to mean temperature incubation (Burgess and Griffin, 1968; Jensen and Reynolds, 1969; Reynolds et al., 1972a,b). More recently, a study found that *Pseudomonas aeruginosa* exhibits respiration rate changes in response to a 2°C DTR associated with daily body temperature changes (Kahl et al., 2022). That such responses can occur under a relatively small DTR suggest that diurnal T fluctuations should be expected to exert a large influence on soil microorganisms where much higher magnitude DTR is found, particularly nearer to the surface. *Bacillus subtilus* also responds to a similar DTR, however it was shown that this organism appears to have a circadian clock controlling its metabolic response once exposed to a 24 h temperature cycle (Eelderink-Chen et al., 2021). Nitrogen fixation rates in soybean nodules, mediated by *Rhizobium* sp., were shown to be correlated with diurnal T change rather than with light cycles associated with photosynthesis in the plant (Weisz and Sinclair, 1988). Fungi, also abundant in soil, have been shown to also have a circadian physiological response to temperature change (Sartor et al., 2019). The presence of circadian rhythms suggests more complex responses to temperature exist among Prokaryotes and suggest that similar differential responses to temperature likely exist with different populations in soil.

**Table 1:** Examples of bacterial- and fungal culture response to diurnal temperature change.

Number	Short description	Ref.
1	Three fungal pathogens tested at different median T and at DTR of 4° and 8°C based on observed DTR at 5 cm in soil in New South Wales. Some responded with faster rates under DTR and others with slower rates under DTR.	Burgess and Griffin (1968)
2	*Fusarium solani* – faster growth rates with DTR from 60° F–80° F compared to the mean T of 70° F.	Jensen and Reynolds (1969)
3	*Macrophomina phaseoli* – This fungus shown to grow faster with DTRs of 6°, 12° and 18°C compared to growth at the mean T of 25° and 30°C.	Reynolds et al. (1972b)
4	20 *Ceratocystis* strains were tested at constant Temps of 15°, 20°, 25°, 30° and 35°C and diurnal T variations of 6°, 12° and 18°C around mean T of 15°, 20°, 25° and 30°C. Some strains showed positive growth response to larger DTRs, while others showed negative response relative to the mean T.	Reynolds et al. (1972a)
5	Chemostat enrichment experiment: diurnal T (1°C to 16°C sine wave model) compared to constant 8°C (mean T) incubation. DTR chemostat showed higher diversity and higher biomass compared to constant T.	Upton et al. (1990)
6	*Klebsiella aerogenes* – found temperature induced circadian rhythm with DTR of 1° or 3°C around a mean of 35.5°C	Paulose et al. (2019)
7	*Pseudomonas aeruginosa* biofilm – redox metabolism responded to DTR of 2°C indicating possible circadian clock.	Kahl et al. (2022)
8	Fungal species in surface detrital degradation responding to DTRs (5° and 9°C) relative to mean T (3° and 8°C). Higher growth rates found with diurnal T incubation using a realistic surface T change model. Paper notes that using diurnal T incubations is important.	Dang et al. (2009)
9	*Bacillus subtilis* circadian rhythms induced by DTR = 3°C shown to share the canonical properties of circadian clocks: free-running period, entrainment, and temperature compensation.	Eelderink-Chen et al. (2021)

What are the mechanistic drivers of biochemical responses to diurnal T changes? Before considering some logical possibilities, it is important to recognize that over all life history on Earth there have existed diurnal cycles of light and temperature. Given that we know that organisms have evolved circadian clocks to respond to changes in light, it would seem logical that similar mechanisms would have likely evolved in response to diurnal T changes in surface ecosystems. In fact, there is evidence that circadian clocks evolved in Bacteria, Archaea and Eukaryotes prior to the appearance of multicellular Eukaryotic organisms (Géron et al., 2023). The expression of specific genes (*kaiA, kaiB*, and *kaiC*) has been well characterized in regulating circadian clocks in cyanobacteria and Eukaryotic organisms (Pitsawong et al., 2023). Many non-photosynthetic bacteria are known to contain homologs of KaiC; for example, *Klebsiella aerogenes* has genetic homologs to these genes and has been shown to have a circadian clock triggered by diurnal T change (Paulose et al., 2019). *Bacillus subtilus* also expresses a clear circadian clock in response to T, even though this organism’s genome does not contain any *kai* gene homologs (Eelderink-Chen et al., 2021). It is also quite likely that more primitive adaptive genetic responses to DTR, other than established circadian clock mechanisms, exist among Prokaryotic species. One such mechanism has been coined temporal mutualism, in which the community of microorganisms are tuned together to respond to DTR in a unique way that does not occur at constant temperatures (Sartor et al., 2019).

From a biochemical perspective, we note that the impacts of T increase on rates of enzyme-catalyzed reactions still provides an important part of understanding the overall effect on these processes in soil ecosystems. Unfortunately, there is a propensity to explain all soil process rates with this type of enzyme-driven model, even though some of the reported results do not follow a model fit. For example, CO_2_ fluxes (i.e., respiration) are reported to exhibit hysteresis in surface soils when exposed to large diurnal T changes (Adekanmbi et al., 2022; Song et al., 2015; Subke and Bahn, 2010; Zhang et al., 2015). Attempts to calculate Q_10_ values (i.e., the relative rate change in an enzymatic process that occurs with a 10° C change in temperature) in these situations have revealed that the values vary depending on whether the temperature is increasing or decreasing between the minimum and maximum temperatures (Zhu and Cheng, 2011). Such variation should not occur if the effect of temperature change was based solely on enzyme kinetics.

### Surface diurnal T changes are globally substantial

Surface diurnal temperature changes of significant magnitude (i.e., 5°C to 30°C) are a global phenomenon that have occurred over Earth’s entire history. Due to efficient absorption of solar energy, daily temperature changes are even greater within surface soils than observed for daily air temperature changes. This phenomenon is clearly described in Rudolf Geiger’s book “The Climate Near the Ground,” translated from German in 1950 (Geiger, 1950). Several studies conducted in the past 60 years noted the nature of diurnal temperature change in surface soils (Gupta and Gupta, 1982; Blackmer et al., 1982; Nadi and Lai, 1984; Cochrane and Baker, 1985; Maljanen et al., 2002; Sun et al., 2006a,b; Jacobs et al., 2008; Subke and Bahn, 2010; Lei et al., 2010). Mathematical predictive models of subsurface T that considered soil surface maximum temperatures exceed air temperature maxima have been around for some time (Parton and Logan, 1981) and updated models which also include shallow surface water are only recently available (Lei et al., 2010; Jacobs et al., 2008). These daily patterns of temperature largely follow the nature of the surface environment, such as soil type, water content, and vegetation cover. Depending on the latitude, the nature of the diurnal temperature change varies seasonally. For example, in temperate latitudes like the agricultural Midwest, similar large surface DTRs occur from April through October. A significantly large historical database of surface temperatures exists, and a recent study from China noted that the DTR gradually decreased with global climate change (Liu et al., 2022). What the authors do not point out, but is clear from the data shown, is that the magnitude of surface DTR all across China for all seasons ranges from 10°C to 40°C. Within the last 10 years, several studies have used satellite-based remote sensing to determine surface temperature characteristics (maximum T, minimum T, and DTR) in many parts of the world (Wang and Prigent, 2020; Liu et al., 2022; Wang et al., 2023). These types of studies have shown differences between the DTR in western United States compared to the eastern part of the country, although it is still of significant magnitude in both areas (Sun et al., 2006b).

Maximum surface temperatures on Earth often appear quite extreme. In temperate regions during the summer, high temperatures (> 50°C) occur frequently in the upper soil layers (top 5 cm), markedly higher than the corresponding ~35°C average maximum air temperatures. Satellite data supports this observation for agricultural soils, but also indicates the hottest non-volcanic surfaces occur in desert areas where soil temperatures exceed 70°C, greater than 25°C above the air temperature (Mildrexler et al., 2011). These high surface temperature conditions can provide transient niche conditions for thermophilic bacteria to grow more optimally (Myers, 1975; Santana and Gonzalez, 2015). These extreme high temperatures are always associated with large DTRs, mainly due to the radiative cooling that occurs at night (Gonzalez et al., 2015).

**Figure 1:**
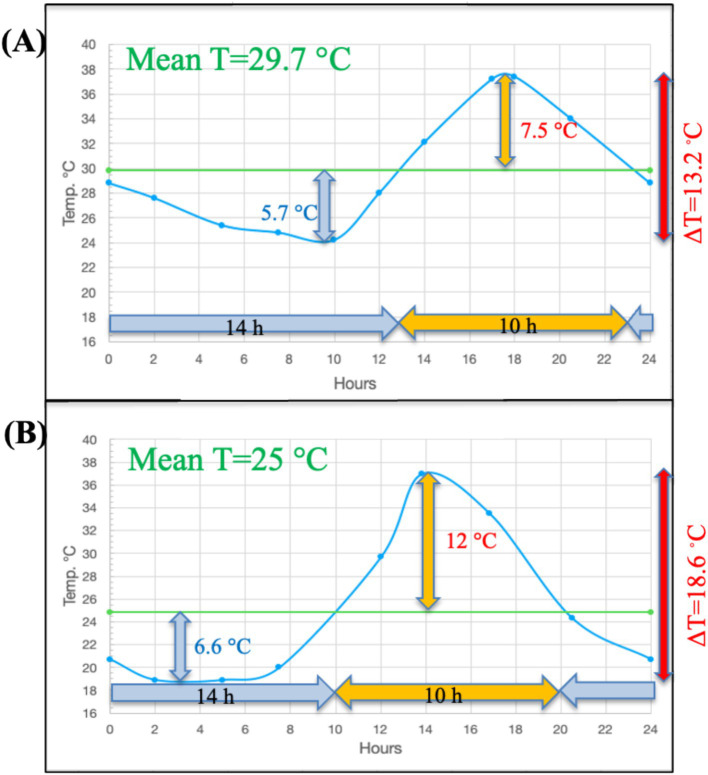
The typical asymmetry of diurnal temperature changes in tropical and midwestern U.S. surface soils for **(A)** the Lajas NEON site in Puerto Rico at 2 cm depth over a 24 h period in June 2018 and **(B)** the Havana, IL agricultural field site at the soil surface in a 24 h period in June 2014 (see Supplementary materials). Note that the period of time the temperature stays above the mean temperature is always less than the time spent below the mean T, and the temperature change between the mean temperature and maximum temperature is always greater than the difference between the mean temperature and the minimum. Note the overall shape of the temperature change curve is fairly similar to a sine-wave model.

**Figure 2:**
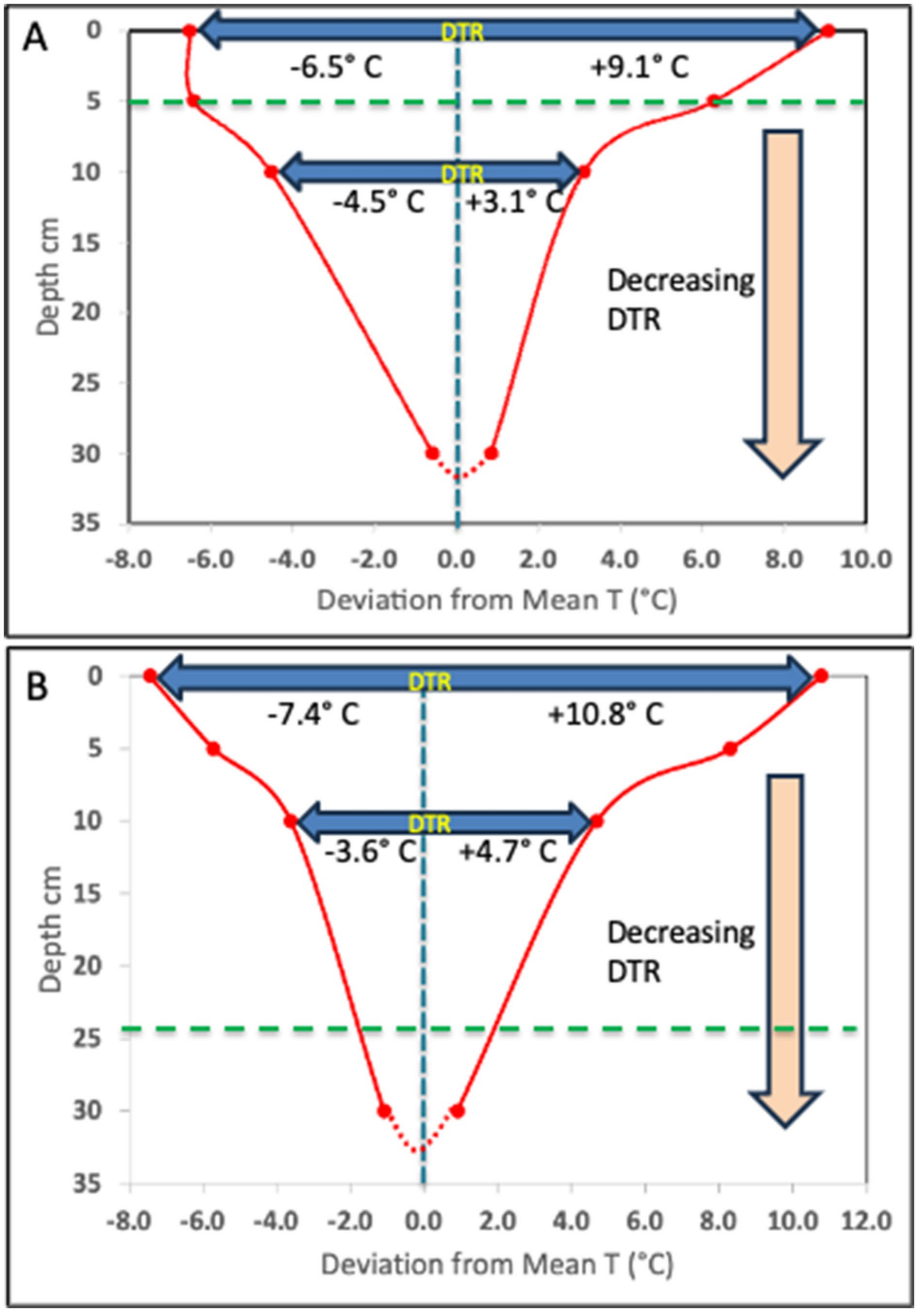
Decrease in DTR with depth, from 15.6° C to 1.4° C and 18.2°C to 2.0°C, as observed in two Illinois agricultural field sites (see Supplementary materials); Urbana **(A)** and Havana **(B)**, respectively. The data plotted reflect the deviation from the observed mean T of the maximum and minimum T at the surface, 5 cm, 10 cm and 30 cm depth. This pattern of DTR is consistent from April through October every year, regardless of the changing mean T. The horizontal dashed green line indicates the depth of DTR symmetry, when the temperature change above and below the mean are equal.

We illustrate here the pattern of temperature change that can be observed in soil over a diurnal cycle. Surface diurnal temperature change follows a typical pattern in both temperate (Illinois agricultural soil-unpublished data) and tropical (Lajas Experimental Station, Puerto Rico-National Ecological Observatory Network (NEON) data) regions (Figure 1). In both locations, the time below the mean temperature exceeds the time above the mean temperature near the soil surface. The DTR decreases rapidly with soil depth from the surface and in two Illinois agricultural soils was less than 2°C at 30 cm compared to nearly 16°C at the surface (Figure 2 – unpublished data-see Supplementary material). Although the temperature profiles are not identical at these two study sites in Illinois due to differences in soil type, the nature of the DTR with depth remains constant over an extended period of each year from April through September. In addition to the two Illinois site data, this consistent relationship with DTR and depth occurs at many NEON sites (Supplementary Figures S1, S2; Supplementary Table S1) with different ecosystems, latitudes and soil types. In all cases the DTR diminishes to less than 2° C at depths approaching 30 cm. Additional data from several western USA sites, available from the Henry Mount Soil Temperature and Water database, confirms the large surface soil DTR (Supplementary Table S2).

### The nature of the DTR with depth in soil

**Figure 3:**
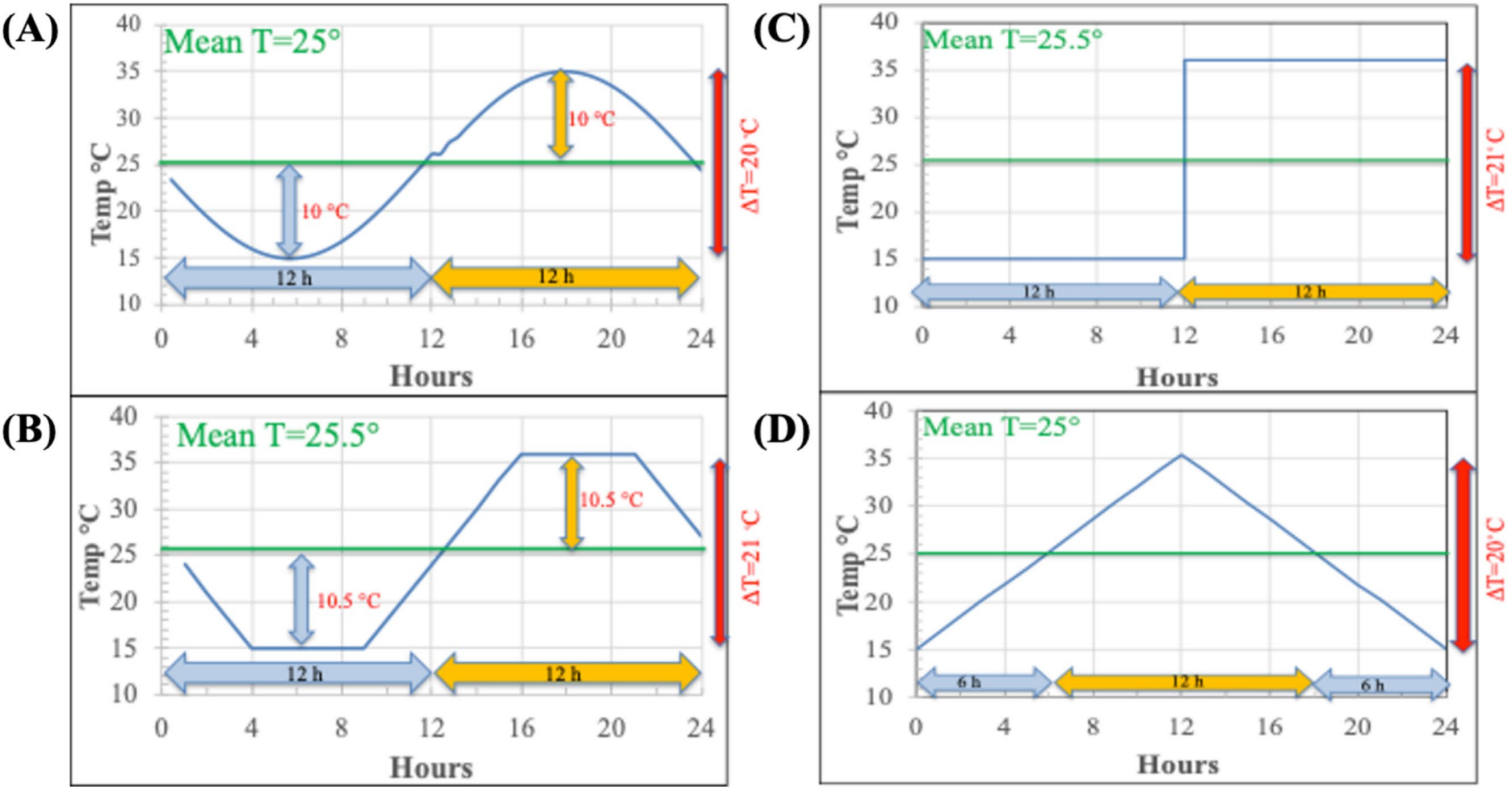
Representative temperature profile models used in previously published diurnal temperature incubation experiments. **(A)** Sinusoidal function of temperature change (e.g., Campbell et al., 1973; Goodroad and Keeney, 1984), **(B)** linear shift function between maximum and minimum temperatures—square wave model (e.g., Campbell et al., 1973), **(C)** a simple maximum/minimum diurnal model (e.g., Thiessen et al., 2013), and **(D)** continuous rate of rising and falling temperature change between the maximum and minimum temperatures (Li et al., 2017). Note here that the diurnal temperature change depicted is a symmetrical cycle with regards to both the mean temperature and the period of time above and below the mean temperature. This is in contrast to the actual diurnal temperature changes that occur in soil (Figure 1).

All previous studies that report evaluating DTR in their experiments typically used oversimplified temperature programs as proxy for true soil diurnal T cycles. With the possible exception of the sine wave model, these do not come close to replicating actual diurnal temperature change in surface soils (Figures 1, 3) (Campbell et al., 1973; Goodroad and Keeney, 1984; Thiessen et al., 2013; Akbari and Ghoshal, 2015; Bai et al., 2017; Li et al., 2017). Many of the temperature models used in other studies present several issues that could confound the interpretation of results. In these temperature programs, the ∆T is always symmetrical relative to time (12 h above-and 12 h below the mean T) and relative to the mean T (equal degrees above and below the mean). Some of the models also set the minimum-and maximum temperatures as constant for extended periods of time (e.g., 6 h–12 h at each temperature). In reality, the diurnal temperature changes exhibit asymmetrical behavior relative to time and the mean T (Figure 1). At the Lajas NEON site in Puerto Rico and two agricultural field sites in Illinois, the surface temperature fell below the mean 14 h per day and above the mean 10 h per day in June (Figures 1, 2). As a result, the maximum surface temperature difference with the mean T is always greater than the difference between the minimum temperature and the mean T. Figure 2 shows, however, that at the two Illinois sites there is a depth in the soil profile where the time above the mean T and time below the mean T are equal. This symmetrical DTR occurs at different depths (5 cm and 25 cm) and is probably due to the soil texture differences (silty loam vs. sandy) between the two sites. At these depths a sine wave model of diurnal T change would be a fairly close proxy to what occurs at that horizon, but this symmetrical DTR would be unique to that depth and the specific soil type. Note in Figure 2A, once the maximum and minimum T have equal deviation from the mean T (at 5 cm), the minimum T becomes greater than the deviation with the maximum T as the depth increases. At these greater depths the time above the mean must be larger than the time below the mean T.

### Observations of microbe-mediated process rates in response to DTR in surface ecosystems

Soil microbial processes (e.g., mineralization rates and N-cycle rates) have been shown to respond to diurnal temperature changes in ways not predicted by the mean temperature (Table 2). Studies over the last half century have clearly shown this relationship and several of them have even noted the diurnal T change, however none of them have considered the possible evolutionary adaptation to DTR itself as something that could at least partially explain the observations made.

**Table 2:** Examples of surface process responses to diurnal temperature change in field and laboratory studies.

Number	Short description	Ref.
1	This study shows that nitrification rates in soil were greater with a sine-wave diurnal T incubation with a mean of 8.5°C (3° to 14°C, DTR = 11°C), than at the mean only or with a square wave function diurnal T incubation. *Suggest future studies should use diurnal T change*.	Campbell et al. (1973)
2	Paper notes N_2_O flux in field study varies diurnally in conjunction with surface DTR.	Ryden et al. (1978)
3	Field study in grass sward that shows diurnal N_2_O flux associated with observed DTR of 8°C (6°–14°C) at 3 cm depth.	Denmead et al. (1979b)
4	This is a field study with a flooded rice field that noted N_2_O flux correlated with diurnal T change in the surface flooded water.	Denmead et al. (1979a)
5	Iowa field study shows that N_2_O flux varies diurnally with change in T at 2 cm depth. DTR is ~15°C at this depth. Shade experiment with little DTR showed no diurnal N_2_O flux variance. Recorded DTR at 5 cm to be 10°C or higher from April through October.	Blackmer et al. (1982)
6	Study showing that N_2_O flux under diurnal T incubation (sine wave 15°–25°C) is different than what is observed at the mean temperature of 20°C.	Goodroad and Keeney (1984)
7	Study shows that N-fixation rates in soybean nodules respond to diurnal T changes (19°-29°C) in contrast to constant mean T and in contrast to photosynthesis.	Weisz and Sinclair (1988)
8	Study reports gas fluxes from a peat field site and notes diurnal variation in CO_2_ and N_2_O fluxes associated with the air T variation.	Maljanen et al. (2002)
9	Greenhouse study with mulch over mineral soil. DTR of 8°C recorded and shown to be correlated with increased N_2_O and CO_2_ flux.	Flessa et al. (2002)
10	This study observed that soil CO_2_ flux exhibited significant diurnal changes that were highly correlated with the soil surface T changes.	Nakadai et al. (2002)
11	Study showed that soils incubated at constant T (20°C) exhibited different respiration Q10 values than those incubated under diurnal T conditions (15° to 25°C).	Zhu and Cheng (2011)
12	Study presents testing of 3 different soils under different T regimes (15°, 20°, and 25°C and a diurnal 15°-25°C). They show that the DTR incubation yields different respiration rates than the average of 20°C.	Ci et al. (2015)
13	Study compares constant low and high T incubations in soil to diurnal variation 10° to 30°C. They note that the Q10 of the DTR incubation exceeds that observed in the fixed T incubations.	Bai et al. (2017)
14	Study demonstrates that respiration rates in soil were higher with a diurnal T incubation (5°–15°C) than with a mean T incubation (10°C). The diurnal T rates were statistically the same as those observed at the maximum T.	Adekanmbi et al. (2022)

Although previous studies did not use accurate models of soil diurnal temperature changes, several of these demonstrate rates of processes did differ with varying temperatures regimens compared to static temperature incubations (Table 1). More than five decades ago, Campbell et al. (1973) showed that diurnal temperature incubations using a sine-wave model (Figure 3A) with a DTR of 20°C exhibited higher ammonification and nitrification rates compared to incubations at a fixed mean T. When they used a square-wave model (Figure 3B) for the diurnal temperature incubation, they did not observe a clear distinction in rates compared to that under fixed mean T. Such differences between results obtained using the square-wave T versus the sine-wave T incubation further serve to demonstrate not only the importance of temperature variation on microbial activities, but the pattern by which temperature varies also matters. Using a sinusoidal pattern of temperature, another study showed that the highest rates of N_2_O emission corresponded to the maximum temperature (Goodroad and Keeney, 1984). The paper emphasizes that diurnal variation in temperature adds uncertainty to quantifying N_2_O flux from soil. In another study, N_2_O emission rates from a field varied with the diurnal temperature change and corresponded to peak nitrification rates (Denmead et al., 1979a). In that study, however, the rates of N_2_O emission were higher at the mean temperature when the temperature is increasing and lower at the mean temperature when it is decreasing. Recent studies have shown similar hysteresis patterns in rates of respiration or N_2_O emissions from soils relative to daily temperature change in both laboratory and field settings (Li et al., 2017; Pingintha et al., 2010). Although these studies show effects of diurnal temperature change on rates of respiration and N_2_O emissions, they have all focused on near surface soils and comparisons were not made with soil from deeper horizons. Further, the apparent failure of many contemporary studies to acknowledge precedence established by a significant body of literature that address the effects of temperature on soil microbial processes in hindsight serves to illustrate perhaps how disciplinary silos have likely influenced current research approaches.

### Microbial community changes with soil depth: evidence of DTR effect

Only a couple of studies have noted the significant change in microbial community structure with depth, however none of these have attributed this directly to the DTR (Eilers et al., 2012; Li et al., 2022). Eilers et al. (2012) found that the microbial beta-diversity in the near surface was universally different from the community composition at depth. The variance in microbial community composition corresponding to depth (shallowest 2–6 cm to deepest 20–165 cm) at nine sites within the Boulder Creek Critical Zone observatory was often greater than the variance across 54 other globally distributed shallow (0–5 cm) microbial community. They attributed the large gradient of microbial community composition along depth to soil moisture or carbon content and did not consider temperature or DTR. In contrast, Li et al. (2022) do mention surface DTR as a factor influencing the shallow community relative to the deep. Their study, however, uses 0–10 cm soil to represent the shallow soil fraction which we deem likely to encompass a larger zone of soil than would specifically describe the surface microbial community. They further attribute only about 25% of the surface microbial community assemblage to deterministic drivers, which seems inconsistent with the notion that DTR exerts a strong selective force. A metagenomic sequence analysis of DNA extracted from soil samples taken from 0 to 5 cm and 20 to 30 cm depths from our two Illinois sites shows clear differentiation between the microbial communities from these two depths (Orellana et al., 2018, 2019). Community analysis data collected with depth at these two Illinois agricultural sites taken over 4 years shows that DTR alone or in combination with other environmental temperature or chemical variables yielded the highest correlations to the differences in patterns of beta-diversity observed (Supplementary Figure S3; Supplementary Tables S4, S5).

Some studies have suggested that the presence of thermophilic microorganisms in surface soils may account for the community composition differences observed with depth and explain differences in biogeochemical process rates. One study identified DTR as a primary driver of the unique microbial community found in surface soils but attributed this to the presence of thermally tolerant microbial populations, even true thermophiles (temperature range + 46 ° to +75°C) (Hu et al., 2019). Santana and Gonzalez (2015) actually demonstrate clear thermophilic microbial activity in surface soils experiencing a large DTR and a high maximum T (>45°C). We suggest that respiratory processes in surface soils likely include a contribution from thermophiles, however their contribution has also been overlooked in most previous studies. Thermophilic temperatures in temperate climates, however, only occur in the summer, while large surface DTR are prevalent from April through October and in higher latitudes where soil surface temperatures never reach thermophilic ranges. This suggests that in addition to physiological responses intrinsic to being thermophilic, DTR itself likely is a selective force to which the microbial populations in a community can adapt.

### Adaptation to DTR—experimental evidence and potential mechanisms

Although several previously published studies have recognized daily near-surface changes in soil temperature, most have not considered this as an explicit ecological factor that potentially selects for microbial populations that have adapted to the DTR that is present in near surface soils. Few published articles have suggested that soil processes should be evaluated under diurnal temperature change conditions and should be included in future studies; a decades-long gap since one article published over 50 years ago (Campbell et al., 1973) belies the serious oversight existing in modern studies regarding temperature impacts on biological processes. While Adekanmbi and Sizmur (2022) emphasize the importance of including surface DTR in soil experiments, they attribute the biological effect to enhanced organic polymer degradation as the primary mechanism affected by temperatures above the mean T that leads to the increased CO_2_ flux. They do not consider any possible direct microbial or fungal genetic adaptation to DTR as possibly contributing to their experimental observations (Adekanmbi et al., 2022). In an analysis to measure correlations between the patterns of environmental variables and microbial communities corresponding to soil depth (see Supplementary Figure S3), surface DTR emerged among the strongest correlated factors (Supplementary Tables S4, S5). This suggests that further testing the importance of DTR alone or in combination with other variables in driving microbial community structure and function is warranted. Results from a microcosm experiment that compared the response to large DTR compared to a mean T in soils from 0 to 5 cm depth with soils from 30 cm depth clearly show a different respiratory response in shallow vs. deep soils. Deep soils showed no difference in CO_2_ or N_2_O emissions regardless of a large DTR or mean T incubation regime (Supplementary Figure S4). Thus, there appears to be more to the DTR response than just enhanced organic polymer degradation. Although temperature change effects on microbial processes have been more commonly addressed in recent studies, the majority of these use static temperature settings instead of dynamic changes that occur under actual soil diurnal temperature change. Li et al. (2022) suggest that creating experimental conditions that closely match actual environmental temperature conditions is important. They also note that only one study of 46 referenced since 1993 evaluated DTR in their temperature change effect experiments (Zhu and Cheng, 2011), a detail relegated to supplemental information. Although this information provided here may not be exhaustive, it indicates a prevalence of studies that have not considered DTR in their experiments. Even in experiments not evaluating temperature change, there has been a continued tendency to assume the daily mean T in soil is sufficient to evaluate biogeochemical processes.

### Discussion and historical perspective on soil activity response to T change

Most of the previous studies in soil consider changes in temperature to impact rates of processes based on biochemical kinetic theories that are often summarized as Q_10_ values (i.e., the proportional change in rate with a 10°C change in temperature). Typical Q_10_ values of around 2.0 are often assigned to soil respiration activity even though many studies have shown Q_10_ values decrease as the temperature increases (Oertel et al., 2016). Some studies have even shown that 29–55% higher Q_10_ values are obtained when using variable diurnal temperature incubation with near surface soils compared to static incubations at the minimum and maximum temperatures (Bai et al., 2017). Another observation of Q_10_ in the context of a diurnal temperature change is that two different values have been reported for the same temperature range depending on whether the temperature is increasing or decreasing (Zhang et al., 2015). This hysteresis seems inconsistent with only a simple temperature change model impact on reaction rates.

Based on the observations of inconsistencies in reported Q_10_ values relative to the expected response according to Arrhenius enzyme temperature effects, it seems logical to consider other factors that contribute to what is observed with a large surface DTR. These include genetic adaptation, such as the presence of circadian clock genes, and the potential activity of thermophilic organisms, that are active at temperatures above 45°C. Many studies have now established the existence of circadian clock mechanisms that respond to temperature in both bacteria and fungi (Table 1). Some of these experimental studies show the absence of known circadian clock genes, yet still exhibit the expected physiological response for having such a genetic mechanism, suggesting as yet unrecognized circadian mechanisms may also be in play. Another possible genetically related response relates to the potential existence of temporal mutualism, in which populations in the community act together in a unique way in response to diurnal temperature change that differs from the activity measured at the mean T. In addition, surveys of genomes and metagenomes have established the existence of circadian clock gene homologs in over 3,000 Bacteria and Archaea, most of which are not photosynthetic cyanobacteria (Géron et al., 2023). This genetic record suggests that these genes are old in the evolutionary record, going back to the last common ancestor. Possible expectations of activities in soils due to either a circadian clock or temporal mutualism would be a large deviation from the expected Q_10_ response or no Q_10_ response. One recent study seems to support one of these expectations, where soil CO_2_ flux rates were measured several times a day for multiple days (Bond-Lamberty et al., 2016). Remarkably, even though the DTR at the surface was 10°C or larger, there was no change in the CO_2_ flux (respiration) rate from one of soils tested. This supports the possibility of a circadian response that controlled a relatively constant respiration rate despite temperature variation.

While there have been previous soil studies attempting to address diurnal temperature variation and its impact on N-cycle processes and/or respiration rates, all of these studies typically used oversimplified temperature programs as proxy for true soil diurnal T cycles. Many of the temperature models used in other studies present several issues that could confound the interpretation of results. In these temperature programs, the DTR is often symmetrical relative to time (12 h above-and 12 h below the mean T) and relative to the mean T (equal degrees above and below the mean) (Figure 3). In addition, some of the models set the minimum-and maximum temperatures as constant for extended periods of time (e.g., 6 h at each temperature). In reality, the diurnal temperature changes exhibit asymmetrical behavior relative to time and the mean T (Figures 1, 2; Supplementary Figures S1, S2). In addition, we observe these features in data collected at the 2 cm depth of the NEON sites (Jornada, Sterling and Konza) in both April and June (Supplementary Figure S2). It is also apparent that near surface soils spend varied amounts of time near the maximum or minimum temperatures, ranging from <2 h to ~4 h, respectively (Figure 1). We note that this asymmetry changes with depth and that the nature of the change depends on the soil type. For any soil type there is depth where the time above the mean becomes equal to the time below the mean (Figure 2). At this depth, the DTR exhibits symmetry like that of a sine-wave function, which might explain why experiments done using such a function to mimic DTR have been successful (Campbell et al., 1973). Since temperature poses a critical influence on microbial activity, the actual characteristic of soil temperature changes at any specific depth is likely of paramount importance. Indeed, our ability going forward to accurately predict biological temperature responses should focus attention on generating data to better inform model equations commonly used to estimate biological processes. We anticipate the shift away from soil experiments conducted at constant temperatures or with imprecise diurnal temperature cycles that do not follow *in situ* conditions will improve our ability to examine the underlying nature of temperature effects on important microbial processes in soils. To this end, one suggestion could be the use of plant growth chambers where programmed diurnal temperature and light cycles for incubation to better serve as proxy for soil temperature dynamics.

Although previous studies did not use accurate models of soil diurnal temperature changes, several of these demonstrate rates of processes did differ with varying temperatures regimens compared to static temperature incubations (Table 2). For example, a study by Campbell et al. (1973) showed a clear response to DTR when using a sine-wave T change model and no response when using a square wave T change model (Figure 3). Such differences between results obtained further serve to demonstrate the importance of accurately replicating temperature change patterns that exist in different surface ecosystems.

## Conclusion

**Figure 4:**
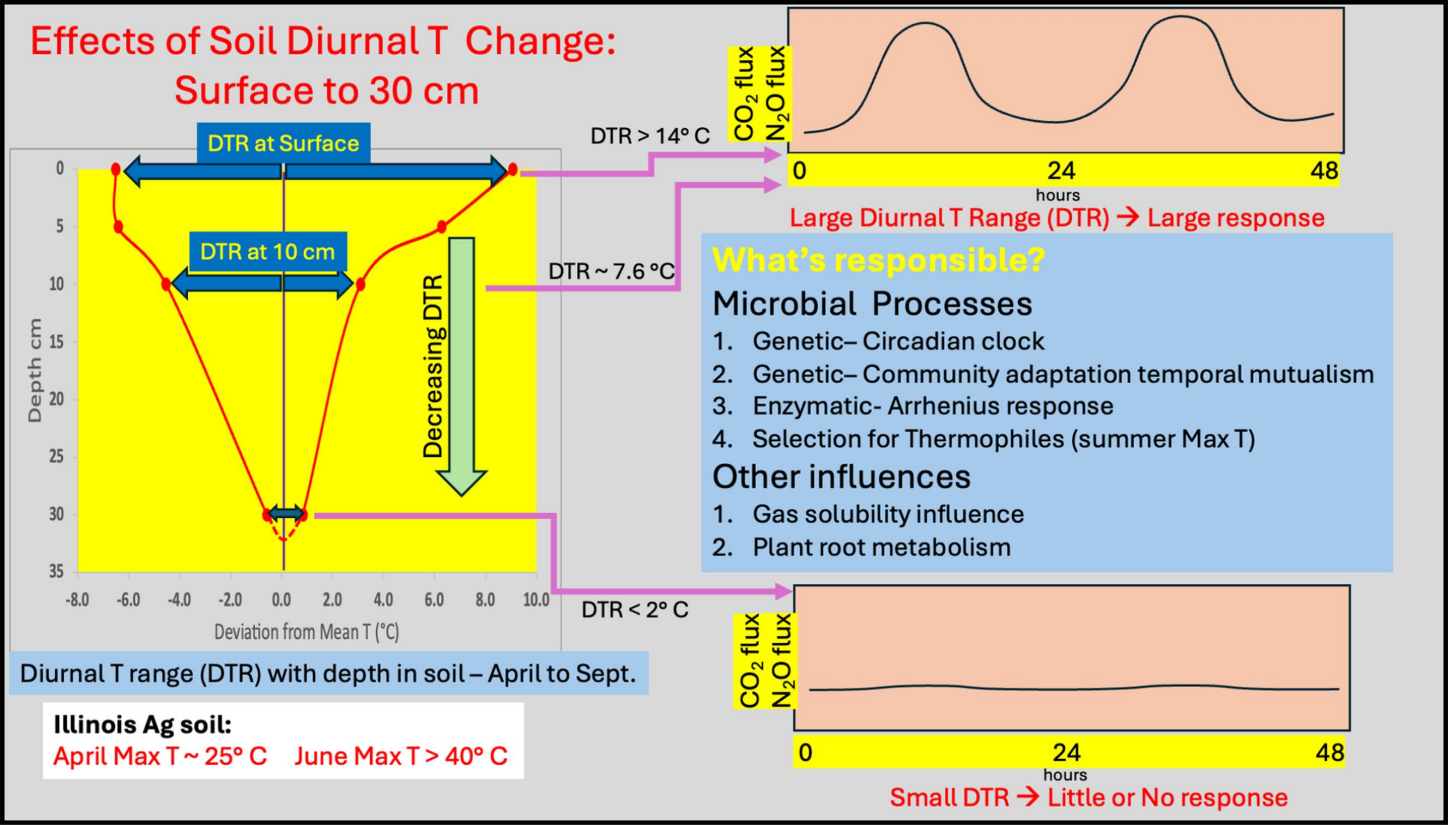
Diagram of DTR with depth around the mean for typical silty loam soil as observed from April to September. From the surface to 10 cm depth the DTR is large enough to yield a differential activity response; here shown as model CO_2_ or N_2_O fluxes. This differential activity response is minimized at depths of 30 cm or greater where the DTR is less than 2°C. Possible mechanisms contributing to this activity response are listed. All may be important and all support the need to evaluate microbial mediated activities in soil using realistic diurnal temperature variation.

A careful overview of the literature establishes that microorganisms (Bacteria, Archaea and Fungi) often exhibit a differential activity response to diurnal temperature change. Through our own observations, we also show the nature and magnitude of DTR with depth along with the associated microbial community composition. This provides further support of recent literature that the magnitude of surface DTR is globally significant and has been for all of life history on Earth. We also note that the DTR changes rapidly with soil depth, indicating that surface (0–5 cm) microbial community activity and response to DTR is likely to be quite different than the activity observed at depth (e.g., 26–30 cm). Figure 4 provides a conceptual illustration of the DTR changes with depth and its potential impact on microbially mediated process. The possible mechanisms that contribute to the differences in activity are listed and they all support the idea that diurnal change in temperature is important in terms of evaluating CO_2_, N_2_O or even CH_4_ gas flux from soil. Adaptive mechanisms related to genetics, such as the presence of a circadian clock or a community temporal mutualism response have not yet been considered as contributing to microbial processes in surface soils. Even thermophilic microorganisms that thrive at the higher T observed in temperate latitude surface soils in summer are ignored in soil studies.

We advocate the use of temperature incubations in studies that serve to provide a better proxy for near surface soil conditions to measure biological activities that occur in these environments. With more supporting realistic diurnal T studies like this in the future, we can test new hypotheses about microbial communities such as adaptation to the legacy T conditions, possibly due to temporal mutualism, and how this would affect important C and N processes. Genetic adaption related to DTR associated circadian clocks need to be considered, since they have clearly been shown to exist in non-photosynthetic Prokaryotes and fungi. Concurrent with shifting approaches to the study of temperature on microbial processes, molecular-based (DNA-and RNA-based) community characterization together with process measurements will significantly advance our understanding of this important relationship. Such an approach can better yield more accurate data for predictions of microbial processes and improve our understanding of soil microbial ecology, especially with contemporary issues surrounding global climate change and emerging paradigm shifts due to ever-increasing new information about soil microbial communities.

## Data Availability

The original contributions presented in the study are included in the article/Supplementary material, further inquiries can be directed to the corresponding author.

## References

[ref1] AAM (2017) in FAQ: microbes and climate change: Report on an American Academy of microbiology and American Geophysical Union colloquium held in Washington, DC, in march 2016. ed. American Academy of Microbiology FAQ Reports. Available at: https://www.ncbi.nlm.nih.gov/books/NBK513763/30063309

[ref2] Adekanmbi A. A. Shu X. Zou Y. Sizmur T. (2022). Legacy effect of constant and diurnally oscillating temperatures on soil respiration and microbial community structure. Eur. J. Soil Sci. 73:e13319. doi: 10.1111/ejss.13319

[ref3] Adekanmbi A. A. Sizmur T. (2022). Importance of diurnal temperature range (DTR) for predicting the temperature sensitivity of soil respiration. Front. Soil Sci. 2:969077. doi: 10.3389/fsoil.2022.969077

[ref4] Akbari A. Ghoshal S. (2015). Effects of diurnal temperature variation on microbial community and petroleum hydrocarbon biodegradation in contaminated soils from a sub-Arctic site. Environ. Microbiol. 17, 4916–4928. doi: 10.1111/1462-2920.12846, PMID: 25808640

[ref5] Bååth E. (2018). Temperature sensitivity of soil microbial activity modeled by the square root equation as a unifying model to differentiate between direct temperature effects and microbial community adaptation. Glob. Chang. Biol. 24, 2850–2861. doi: 10.1111/gcb.14285, PMID: 29682877

[ref6] Bai Z. Xie H. Kao-Kniffin J. Chen B. Shao P. Liang C. (2017). Shifts in microbial trophic strategy explain different temperature sensitivity of CO_2_ flux under constant and diurnally varying temperature regimes. FEMS Microbiol. Ecol. 93:fix063. doi: 10.1093/femsec/fix063, PMID: 28499007

[ref7] Blackmer A. M. Robbins S. G. Bremner J. M. (1982). Diurnal variability in rate of emission of nitrous oxide from soils 1. Soil Sci. Soc. Am. J. 46, 937–942. doi: 10.2136/sssaj1982.03615995004600050011x

[ref8] Bond-Lamberty B. Bolton H. Fansler S. Heredia-Langner A. Liu C. McCue L. A.. (2016). Soil respiration and bacterial structure and function after 17 years of a reciprocal soil transplant experiment. PLoS One 11:e0150599. doi: 10.1371/journal.pone.0150599 26934712 PMC4775055

[ref9] Buckeridge K. M. Edwards K. A. Min K. Ziegler S. E. Billings S. A. (2020). Short-and long-term temperature responses of soil denitrifier net N_2_O efflux rates, inter-profile N2O dynamics, and microbial genetic potentials. Soil 6, 399–412. doi: 10.5194/soil-6-399-2020

[ref10] Burgess L. W. Griffin D. M. (1968). The influence of diurnal temperature fluctuations on the growth of fungi. New Phytol. 67, 131–137. doi: 10.1111/j.1469-8137.1968.tb05462.x

[ref11] Campbell C. A. Biederbeck V. O. Warder F. G. (1973). Influence of simulated fall and spring conditions on the soil system: III. Effect of method of simulating spring temperatures on ammonification, nitrification, and microbial populations. Soil Sci. Soc. Am. J. 37, 382–386. doi: 10.2136/sssaj1973.03615995003700030021x

[ref12] Ci E. Al-Kaisi M. M. Wang L. Ding C. Xie D. (2015). Soil organic carbon mineralization as affected by cyclical temperature fluctuations in a karst region of southwestern China. Pedosphere 25, 512–523. doi: 10.1016/S1002-0160(15)30032-1

[ref13] Cochrane J. Baker C. R. (1985). Annual and diurnal variations in soil temperatures at Kew, Great Britain. Agric. For. Meteorol. 34, 235–240. doi: 10.1016/0168-1923(85)90023-1

[ref14] Dang C. K. Schindler M. Chauvet E. Gessner M. O. (2009). Temperature oscillation coupled with fungal community shifts can modulate warming effects on litter decomposition. Ecology 90, 122–131. doi: 10.1890/07-1974.1, PMID: 19294919

[ref15] Denmead O. T. Freney J. R. Simpson J. R. (1979a). Nitrous oxide emission during denitrification in a flooded field. Soil Sci. Soc. Am. J. 43, 716–718. doi: 10.2136/sssaj1979.03615995004300040017x

[ref16] Denmead O. T. Freney J. R. Simpson J. R. (1979b). Studies of nitrous oxide emission from a grass sward. Soil Sci. Soc. Am. J. 43, 726–728. doi: 10.2136/sssaj1979.03615995004300040020x

[ref17] Eelderink-Chen Z. Bosman J. Sartor F. Dodd A. N. Kovács Á. T. Merrow M. (2021). A circadian clock in a nonphotosynthetic prokaryote. Sci. Adv. 7:eabe2086. doi: 10.1126/sciadv.abe2086 33523996 PMC7793578

[ref18] Eilers K. G. Debenport S. Anderson S. Fierer N. (2012). Digging deeper to find unique microbial communities: the strong effect of depth on the structure of bacterial and archaeal communities in soil. Soil Biol. Biochem. 50, 58–65. doi: 10.1016/j.soilbio.2012.03.011

[ref19] Flessa H. Potthoff M. Loftfield N. (2002). Greenhouse estimates of CO_2_ and N_2_O emissions following surface application of grass mulch: importance of indigenous microflora of mulch. Soil Biol. Biochem. 34, 875–879. doi: 10.1016/S0038-0717(02)00028-7

[ref20] Geiger (1950). The climate near the ground, vol. 33. Cambridge, MA: Harvard University Press.

[ref21] Géron A. Werner J. Wattiez R. Matallana-Surget S. (2023). Towards the discovery of novel molecular clocks in prokaryotes. Crit. Rev. Microbiol., 1–13. doi: 10.1080/1040841X.2023.2220789 37330701

[ref22] Gonzalez J. M. Portillo M. C. Piñeiro-Vidal M. (2015). Latitude-dependent underestimation of microbial extracellular enzyme activity in soils. Int. J. Environ. Sci. Technol. 12, 2427–2434. doi: 10.1007/s13762-014-0635-7

[ref23] Goodroad L. L. Keeney D. R. (1984). Nitrous oxide production in aerobic soils under varying pH, temperature and water content. Soil Biol. Biochem. 16, 39–43. doi: 10.1016/0038-0717(84)90123-8

[ref24] Gupta G. N. Gupta J. P. (1982). Diurnal variations in moisture and temperature of a desert soil under different management practices. Arch. Meteorol. Geophys. Bioclimatol. Ser. B 31, 133–138. doi: 10.1007/BF02311348

[ref25] Hu A. Nie Y. Yu G. Han C. He J. He N.. (2019). Diurnal temperature variation and plants drive latitudinal patterns in seasonal dynamics of soil microbial community. Front. Microbiol. 10:674. doi: 10.3389/fmicb.2019.00674, PMID: 31001239 PMC6454054

[ref26] Jacobs A. F. G. Heusinkveld B. G. Kraai A. Paaijmans K. P. (2008). Diurnal temperature fluctuations in an artificial small shallow water body. Int. J. Biometeorol. 52, 271–280. doi: 10.1007/s00484-007-0121-8, PMID: 17926069 PMC2668566

[ref27] Jensen K. F. Reynolds P. E. (1969). How two types of fluctuating temperature affect the growth of fusarium solani. Delaware, Ohio: Northeastern Forest Exp. Sta., USDA.

[ref28] Kahl L. J. Eckartt K. N. Morales D. K. Price-Whelan A. Dietrich L. E. P. (2022). Light/dark and temperature cycling modulate metabolic electron flow in *Pseudomonas aeruginosa* biofilms. MBio 13, e01407–e01422. doi: 10.1128/mbio.01407-22 35938725 PMC9426528

[ref30] Lei S. Daniels J. L. Bian Z. Wainaina N. (2010). Improved soil temperature modeling. Environ. Earth Sci. 62, 1123–1130. doi: 10.1007/s12665-010-0600-9

[ref31] Li J. He N. Xu L. Chai H. Liu Y. Wang D.. (2017). Asymmetric responses of soil heterotrophic respiration to rising and decreasing temperatures. Soil Biol. Biochem. 106, 18–27. doi: 10.1016/j.soilbio.2016.12.002

[ref32] Li W. Kuzyakov Y. Zheng Y. Li P. Li G. Liu M.. (2022). Depth effects on bacterial community assembly processes in paddy soils. Soil Biol. Biochem. 165:108517. doi: 10.1016/j.soilbio.2021.108517

[ref33] Liu B. Zhou W. Henderson M. Sun Y. Shen X. (2022). Climatology of the soil surface diurnal temperature range in a warming world: annual cycles, regional patterns, and trends in China. Earth's Future 10:e2021EF002220. doi: 10.1029/2021EF002220

[ref35] Maljanen M. Martikainen P. J. Aaltonen H. Silvola J. (2002). Short-term variation in fluxes of carbon dioxide, nitrous oxide and methane in cultivated and forested organic boreal soils. Soil Biol. Biochem. 34, 577–584. doi: 10.1016/S0038-0717(01)00213-9

[ref36] Mildrexler D. J. Zhao M. Running S. W. (2011). Satellite finds highest land skin temperatures on earth. Bull. Am. Meteorol. Soc. 92, 855–860. doi: 10.1175/2011BAMS3067.1

[ref37] Myers R. J. K. (1975). Temperature effects on ammonification and nitrification in a tropical soil. Soil Biol. Biochem. 7, 83–86. doi: 10.1016/0038-0717(75)90003-6

[ref38] Nadi O. A. O. Lai R. (1984). Diurnal fluctuations in hydro-thermal regime of a tropical alfisol as influenced by methods of land development and tillage systems. Glob. Chang. Biol. 147, 150–158. doi: 10.1002/jpln.19841470203

[ref39] Nakadai T. Yokozawa M. Ikeda H. Koizumi H. (2002). Diurnal changes of carbon dioxide flux from bare soil in agricultural field in Japan. Appl. Soil Ecol. 19, 161–171. doi: 10.1016/S0929-1393(01)00180-9

[ref40] Oertel C. Matschullat J. Zurba K. Zimmermann F. Erasmi S. (2016). Greenhouse gas emissions from soils a review. Chem. Erde-Geochem. 76, 327–352. doi: 10.1016/j.chemer.2016.04.002

[ref41] Orellana L. H. Chee-Sanford J. C. Sanford R. A. Löffler F. E. Konstantinidis K. T. (2018). Year-round shotgun metagenomes reveal stable microbial communities in agricultural soils and novel ammonia oxidizers responding to fertilization. Appl. Environ. Microbiol. 84, e01646–e01617. doi: 10.1128/AEM.01646-17 29101194 PMC5752871

[ref42] Orellana L. H. Hatt J. K. Iyer R. Chourey K. Hettich R. L. Spain J. C.. (2019). Comparing DNA, RNA and protein levels for measuring microbial dynamics in soil microcosms amended with nitrogen fertilizer. Sci. Rep. 9:17630. doi: 10.1038/s41598-019-53679-0, PMID: 31772206 PMC6879594

[ref43] Parton W. J. Logan J. A. (1981). A model for diurnal variation in soil and air temperature. Agric. Meteorol. 23, 205–216. doi: 10.1016/0002-1571(81)90105-9

[ref44] Paulose J. K. Cassone C. V. Graniczkowska K. B. Cassone V. M. (2019). Entrainment of the circadian clock of the enteric bacterium *Klebsiella aerogenes* by temperature cycles. iScience 19, 1202–1213. doi: 10.1016/j.isci.2019.09.007, PMID: 31551197 PMC6831877

[ref45] Pingintha N. Leclerc M. Beasley J. Zhang G. Senthong C. (2010). Assessment of the soil CO_2_ gradient method for soil CO_2_ efflux measurements: comparison of six models in the calculation of the relative gas diffusion coefficient. Tellus B 62, 47–58. doi: 10.1111/j.1600-0889.2009.00445.x

[ref46] Pitsawong W. Pádua R. A. P. Grant T. Hoemberger M. Otten R. Bradshaw N.. (2023). From primordial clocks to circadian oscillators. Nature 616, 183–189. doi: 10.1038/s41586-023-05836-9, PMID: 36949197 PMC10076222

[ref47] Reynolds P. E. Smith W. H. Jensen K. F. (1972a). Effect of constant and fluctuating temperatures on the in vitro growth of *Ceratocystis* species. Trans. Br. Mycol. Soc. 59, 1–9. doi: 10.1016/S0007-1536(72)80035-4

[ref48] Reynolds P. E. Smith W. H. Jensen K. F. (1972b). Influence of constant and fluctuating temperatures on the growth of *Macrophomina phaseoli*. Trans. Br. Mycol. Soc. 58:512-IN15. doi: 10.1016/S0007-1536(72)80103-7

[ref49] Ryden J. C. Lund L. J. Focht D. D. (1978). Direct in-field measurement of nitrous oxide flux from soils. Soil Sci. Soc. Am. J. 42, 731–737. doi: 10.2136/sssaj1978.03615995004200050015x

[ref50] Santana M. M. Gonzalez J. M. (2015). High temperature microbial activity in upper soil layers. FEMS Microbiol. Lett. 362:fnv182. doi: 10.1093/femsle/fnv182, PMID: 26424766

[ref51] Sartor F. Eelderink-Chen Z. Aronson B. Bosman J. Hibbert L. E. Dodd A. N.. (2019). Are there circadian clocks in non-photosynthetic Bacteria? Biology 8:41. doi: 10.3390/biology8020041, PMID: 31121908 PMC6627678

[ref52] Song W. Chen S. Zhou Y. Wu B. Zhu Y. Lu Q.. (2015). Contrasting diel hysteresis between soil autotrophic and heterotrophic respiration in a desert ecosystem under different rainfall scenarios. Sci. Rep. 5:861. doi: 10.1038/srep16779 PMC4663751 26615895

[ref53] Subke J.-A. Bahn M. (2010). On the “temperature sensitivity” of soil respiration: can we use the immeasurable to predict the unknown? Soil Biol. Biochem. 42, 1653–1656. doi: 10.1016/j.soilbio.2010.05.026, PMID: 21633517 PMC2938481

[ref54] Sun D. Kafatos M. Pinker R. T. Easterling D. R. (2006a). Seasonal variations in diurnal temperature range from satellites and surface observations. IEEE Trans. Geosci. Remote Sens. 44, 2779–2785. doi: 10.1109/TGRS.2006.871895

[ref55] Sun D. Pinker R. T. Kafatos M. (2006b). Diurnal temperature range over the United States: a satellite view. Geophys. Res. Lett. 33, 1–4. doi: 10.1029/2005GL024780

[ref56] Thiessen S. Gleixner G. Wutzler T. Reichstein M. (2013). Both priming and temperature sensitivity of soil organic matter decomposition depend on microbial biomass – an incubation study. Soil Biol. Biochem. 57, 739–748. doi: 10.1016/j.soilbio.2012.10.029

[ref57] Upton A. C. Nedwell D. B. Wynn-Williams D. D. (1990). The selection of microbial communities by constant or fluctuating temperatures. FEMS Microbiol. Lett. 74, 243–252. doi: 10.1111/j.1574-6968.1990.tb04070.x

[ref58] van Gestel N. Reischke S. Baath E. (2013). Temperature sensitivity of bacterial growth in a hot desert soil with large temperature fluctuations. Soil Biol. Biochem. 65, 180–185. doi: 10.1016/j.soilbio.2013.05.016

[ref59] Wang X. Prigent C. (2020). Comparisons of diurnal variations of land surface temperatures from numerical weather prediction analyses, infrared satellite estimates and in situ measurements. Remote Sens. 12:583. doi: 10.3390/rs12030583

[ref60] Wang Y.-R. Samset B. H. Stordal F. Bryn A. Hessen D. O. (2023). Past and future trends of diurnal temperature range and their correlation with vegetation assessed by MODIS and CMIP6. Sci. Total Environ. 904:166727. doi: 10.1016/j.scitotenv.2023.166727, PMID: 37673261

[ref61] Weisz P. R. Sinclair T. R. (1988). Soybean nodule gas permeability, nitrogen fixation and dirunal cycles in soil temperature. Plant Soil 109, 227–234. doi: 10.1007/BF02202088

[ref62] Yin C. Fan X. Yan G. Chen H. Ye M. Ni L.. (2020). Gross N_2_O production process, not consumption, determines the temperature sensitivity of net N_2_O emission in arable soil subject to different long-term fertilization practices. Front. Microbiol. 11:745. doi: 10.3389/fmicb.2020.00745, PMID: 32411109 PMC7198778

[ref63] Zhang Q. Katul G. G. Oren R. Daly E. Manzoni S. Yang D. (2015). The hysteresis response of soil CO_2_ concentration and soil respiration to soil temperature. J. Geophys. Res. Biogeo. 120, 1605–1618. doi: 10.1002/2015JG003047

[ref64] Zhu B. Cheng W. (2011). Constant and diurnally-varying temperature regimes lead to different temperature sensitivities of soil organic carbon decomposition. Soil Biol. Biochem. 43, 866–869. doi: 10.1016/j.soilbio.2010.12.021

