# Erratum: Diurnal temperature variation in surface soils: an underappreciated control on microbial processes

**DOI:** 10.3389/fmicb.2025.1586779

**Published:** 2025-03-13

**Authors:** Frontiers Production Office

**Affiliations:** Frontiers Media SA, Lausanne, Switzerland

**Keywords:** surface soil, diurnal temperature, C-mineralization, reaction rates, microbial adaptation

Due to a production error, there was a mistake in the legend for Figure 2 as published. The axes of Figure 2A were erroneously not included. The correct figure appears below.

**Figure 2:**
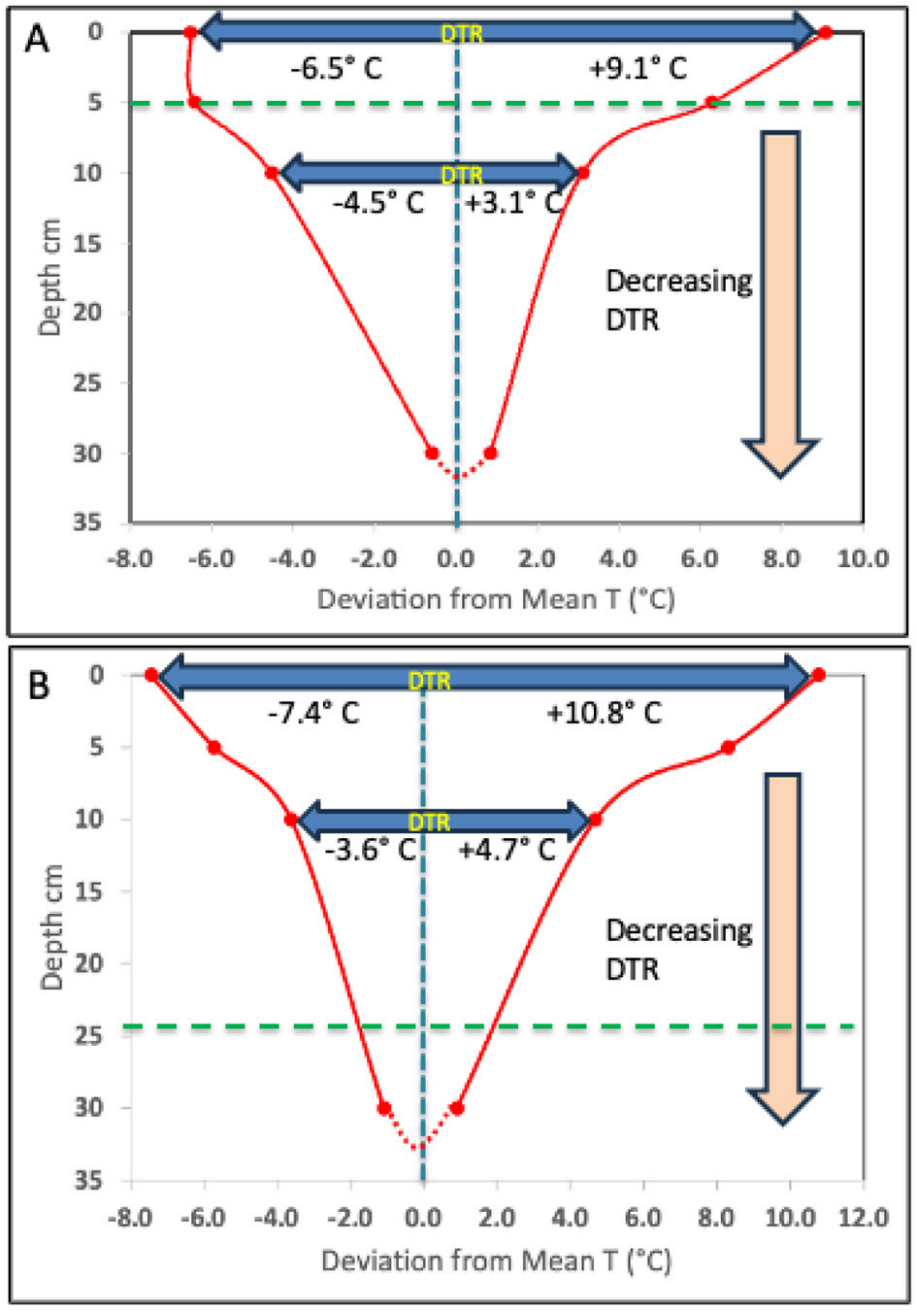
Decrease in DTR with depth, from 15.6°C to 1.4°C and 18.2°C to 2.0°C, as observed in two Illinois agricultural field sites (see Supplementary materials); Urbana **(A)** and Havana **(B)**, respectively. The data plotted reflect the deviation from the observed mean T of the maximum and minimum T at the surface, 5cm, 10cm and 30cm depth. This pattern of DTR is consistent from April through October every year, regardless of the changing mean T. The horizontal dashed green line indicates the depth of DTR symmetry, when the temperature change above and below the mean are equal.

The publisher apologizes for this mistake. The original version of this article has been updated.

