# Supplementary material for: Diurnal temperature variation in surface soils: an underappreciated control on microbial processes
Source: Front Microbiol. 2024 Dec 18;15:1423984. doi: 10.3389/fmicb.2024.1423984 (PMC11688295; doi:10.3389/fmicb.2024.1423984)
Supplement: Supplementary file 1 [file Data_Sheet_1.pdf]

Diurnal Temperature Variation in Surface Soils: an Underappreciated Control on Microbial Processes

By: R. Sanford, J. Chee-Sanford and W. Yang

Supplemental Material

METHODS

Soil Sampling and Soil Temperature Measurements

Two Midwestern U.S. agricultural field sites (each 100 m x 30 m) situated in Illinois (Havana and Urbana) undergoing decades-long corn and soybean rotation were sampled seasonally from 2011 to 2015 to characterize the microbial community composition. The Havana and Urbana field sites represented contrasting soil textures, sandy and silty clay loam, respectively. Temperature data loggers (HOBO TidbiT v2) were deployed at the soil surface, 5 cm, 10 cm and 30 cm depths, with data collected every 15 min. Figure S1 summarizes some of our temperature record for April and June at surface and at 30 cm depth. We collected additional temperature data from several sites at different latitudes from public databases (NEON and Henry Mount Soil Temperature and Water database) showing similar large DTR in shallow soils (2 cm) compared to deeper soils (26 cm) (Figure S2 and Tables S1 and S2). Upon noting the large difference in DTR between the surface and at 30 cm, we conducted a microcosm experiment (see below) to evaluate the response of the microbial community to a diurnal temperature incubation compared to a mean temperature incubation.

Soil cores were taken at three equidistant fixed-location centroids along the length of each field. Seasonal samples of three cores, each partitioned into depth sections were taken from- and 1 m-around each centroid five times a year; April, June, July, September and November in all years. Cores taken in November 2011 were divided into four depth sections 0-5 cm, 5-10 cm, 10-20 cm and 20-30 cm. Subsamples from each core segment were independently used for DNA extraction and subjected to T-RFLP analysis (see below) to establish a community fingerprint. The initial results showed that the 0-5 cm microbial community was significantly dissimilar to the 5-10 cm, 10-20 cm and 20-30 cm communities. In addition, the 20-30 cm community was notably different from the middle depth communities (5-10 cm and 10-20 cm), which could not be separated from each other. Hence, subsequent core samples taken in later years were divided into three segments; 0-5 cm, 5-20 cm and 20-30 cm, which allowed a finer field scale differentiation based on nine core samples taken each time in April, June, July, September and November from the three centroids at each field site from 2012 through 2015 (sample # = 594 per site). Along with samples used for nucleic acid extraction, sample composites from each depth range was used for soil chemical analysis (Table S3). Selected composite samples taken from 0-5 cm and 20-30 cm were used previously in metagenomics and metatranscriptomics studies to characterize microbial taxonomic community structures and functional genes in response to seasonal shifts and soil inputs (Orellana et al., 2018; Orellana et al., 2019). We hypothesized that DTR, large in the 0-5 cm depth, is a strong legacy variable in the corresponding soil depth environment that drives the establishment of stable microbial

community structure. By monitoring these study sites more intensively over several years, we could observe general shifts in their microbial community profiles seasonally in support of the notion that while ephemeral shifts do occur throughout the year due to seasonal agricultural inputs and weather, the communities remain relatively constant in accordance with location and soil depth, regardless of the time of year.

Nucleic Acid Extraction

To obtain soil DNA, individual aliquots (~0.5 g) of Urbana (U) and Havana (H) soil from each depth segment were used for extraction using a phenol-chloroform protocol (Tsai & Olson, 1991). Additional purification of DNA was made with Sepharose 4B (Sigma-Aldrich, St. Louis, MO, USA) gel exclusion, modified from Jackson et al. (Jackson et al, 1997) prior to PCR amplification. All DNA was stored at 20°C for use in this study.

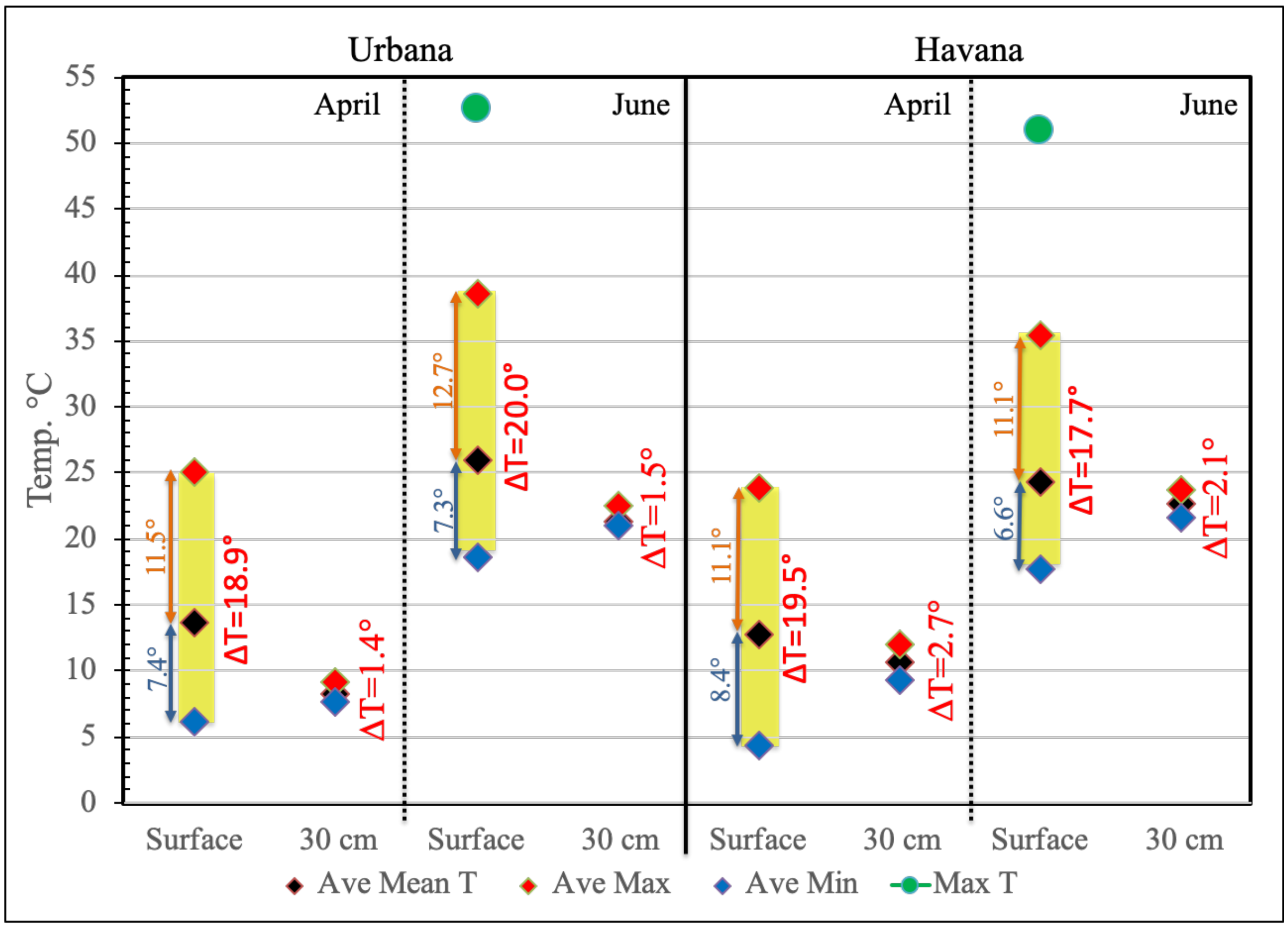

Figure S1. Average temperature variation at Illinois agricultural soil sites in April and June from 2014 - 2017 observed at the soil surface and at 30 cm depth at (A) the Urbana (Drummer/Flanagan silty clay loam) and (B) Havana (Bloomfield sand) agricultural sites in Illinois. At both seasonal time points for each site, significant diurnal ΔTs are observed at the surface even though the mean temperatures are different. Note that the difference between the mean T and the maximum T is greater in all cases than the difference between the mean T and the minimum T.

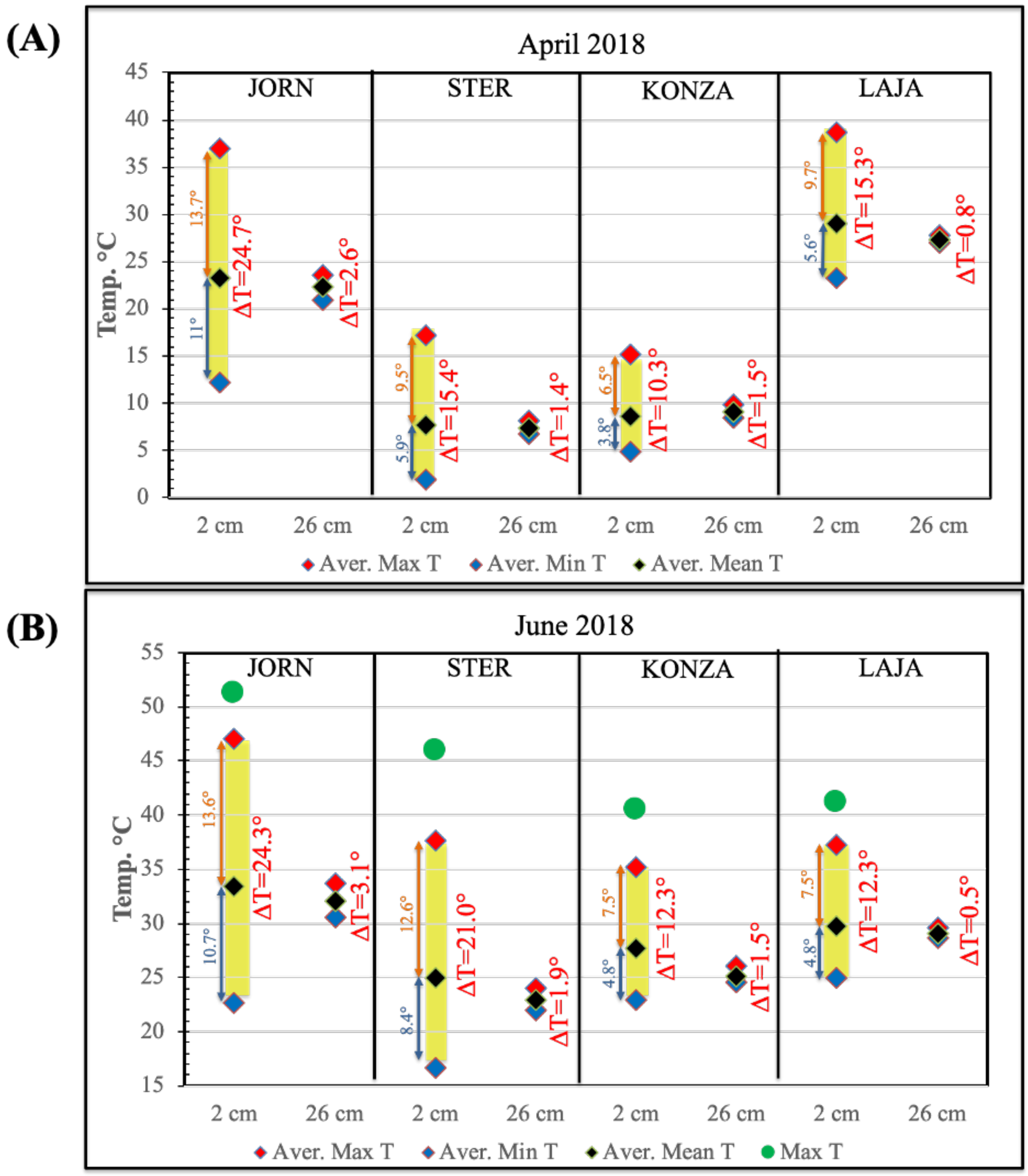

Figure S2. Average temperature variations observed at soil depths of 2 cm and 26 cm at different NEON sites in (A) April and (B) June of 2018. JORN is the Jornada NEON site, STER is Sterling, CO NEON site, KONZA is the Konza Prairie Biological Station NEON site in Kansas and LAJA is the Lajas Experimental Station NEON site in Puerto Rico. At both time points significant diurnal ΔTs are observed at 2 cm depth even though the mean temperatures are different. Note that the difference between the mean T and the maximum T at 2 cm is greater in all cases than the difference between the mean T and the minimum T.

Table S1. Summary of soil temperatures in °C recorded at 2 cm depth in April and June of 2018 from different NEON sites in the United States.

Site	Location	Year	Soil Depth	April				June				
				Mean Max T	Mean Min T	Mean T	Mean DTR	Mean Max T	Mean Min T	Mean T	Mean DTR	Max T
NEON-JORN	Jornada Experimental Range, NM Desert scrub and grassland	2018	2 cm	36.97 *+/- 1.02*	12.24 *+/- 1.16*	23.23 *+/- 0.78*	24.73 *+/- 1.51*	46.92 *+/- 1.25*	22.59 *+/- 1.06*	33.32 *+/- 0.94*	24.32 *+/- 1.48*	51.2
NEON-STER	North Sterling, CO Agricultural	2018	2 cm	17.25 *+/- 2.02*	1.87 *+/- 1.24*	7.74 *+/- 1.40*	15.37 *+/- 1.82*	37.52 *+/- 2.21*	16.55 *+/- 1.23*	24.89 *+/- 1.46*	20.97 *+/- 1.69*	45.82
NEON-KONZ	Konza Prairie Biological Station, KS Native Tallgrass Prairie	2018	2 cm	15.19 *+/- 2.10*	4.86 *+/- 1.67*	8.66 *+/- 1.78*	10.33 *+/- 1.39*	35.18 *+/- 2.09*	22.89 *+/- 1.04*	27.64 *+/- 1.49*	12.29 *+/- 1.42*	40.37
NEON-LAJA	Lajas Experimental Station, PR Agricultural-Grazing	2018	2 cm	38.61 *+/- 0.92*	23.32 *+/- 0.41*	28.95 *+/- 0.39*	15.28 *+/- 1.17*	37.17 *+/- 0.61*	24.89 *+/- 0.24*	29.73 *+/- 0.24*	12.27 *+/- 0.68*	41.07
NEON-DEJU	Delta Junction, AK Srubby Evergreen forest	2018	2 cm	-1.42 *+/- 0.34*	-1.74 *+/- 0.38*	-1.56 *+/- 0.35*	0.32 *+/- 0.08*	13.64 *+/- 0.55*	5.74 *+/- 0.41*	9.21 *+/- 0.39*	7.90 *+/- 0.51*	23.69
NEON-SOAP	Soaproot Saddle, CA Sierra National Forest	2018	2 cm	17.92 *+/- 0.55*	8.56 *+/- 0.38*	12.00 *+/- 0.40*	9.28 *+/- 0.45*	25.34 *+/- 0.72*	13.95 *+/- 0.41*	18.64 *+/- 0.46*	11.40 *+/- 0.63*	35.66
NEON-NOGP	Northern Great Plains Research Lab, ND Native Grassland	2018	2 cm	13.89 *+/- 0.72*	3.08 *+/- 0.68*	7.69 *+/- 0.70*	10.81 *+/- 0.54*	25.94 *+/- 0.46*	15.89 *+/- 0.28*	19.84 *+/- 0.29*	10.05 *+/- 0.49*	33.27

Table S2. Summary of soil temperatures recorded at 5 cm and 2 cm depth from Henry Mount Soil Temperature and Water database.

Site	Year		April Soil Mean T	April Soil Mean Max T	April Soil Mean Min T	April Soil Mean ΔT	June Soil Mean T	June Soil Mean Max T	June Soil Mean Min T	June Soil Mean ΔT
Deer-Hunter	2018	5 cm depth	15.78	18.03	14.05	**3.98**	18.15	21.00	15.91	**5.10**
CA		95% Conf	*+/- 0.34*	*+/- 0.45*	*+/- 0.65*	*+/- 0.79*	*+/- 0.21*	*+/- 0.43*	*+/- 0..36*	*+/- 0.62*
ABCO Cave mnt	2018	5 cm depth					13.57	17.38	10.20	**7.17**
NV		95% Conf					*+/- 0.85*	*+/- 0.94*	*+/- 0.81*	*+/- 0.23*
ARMENDARIS MOUNTAIN	2015	5 cm depth	15.19	20.75	10.55	**10.20**	24.99	32.10	19.12	**12.98**
NM		95% Conf	*+/- 0.58*	*+/- 0.89*	*+/- 0..65*	*+/- 0.98*	*+/- 0.51*	*+/- 0.92*	*+/- 0.79*	*+/- 1.28*
TURTLE PENS	2016	5 cm depth	18.90	24.44	14.14	**10.30**	29.26	35.22	24.12	**11.10**
NM		95% Conf	*+/- 0.64*	*+/- 0.84*	*+/- 0.78*	*+/- 1.01*	*+/- 0.77*	*+/- 0.97*	*+/- 0.85*	*+/- 0.83*
DELHEW	2018	5 cm depth	ND*	0.50	0.24	**0.26**	ND*	17.69	9.84	**7.85**
NV		95% Conf		*+/- 0.27*	*+/- 0.03*	*+/-0.25*		*+/- 0.78*	*+/- 0.76*	*+/- 0.36*
White_Mnts_CA_GR3-T3	2018	1 cm depth	ND*	12.25	11.10	**1.15**	ND*	22.17	21.67	**0.50**
CA		95% Conf		*+/- 0.94*	*+/- 0.97*	*+/- 0.29*		*+/- 0.99*	*+/- 0.96*	*+/- 0.13*
White_Mnts_CA_WP5-I3	2018	1 cm depth	ND*	19.84	9.98	**9.85**	ND*	32.54	19.94	**12.60**
		95% Conf		*+/- 1.41*	*+/- 1.11*	*+/- 1.30*		*+/- 0.94*	*+/- 0.84*	*+/- 0.53*
White_Mnts_CA_WP6-I4	2018	1 cm depth	ND*	24.03	6.18	**17.86**	ND*	43.47	15.76	**27.71**
		95% Conf		*+/- 2.38*	*+/- 1.36*	*+/- 2.08*		*+/-1.48*	*+/- 0.88*	*+/- 1.13*
White_Mnts_CA_study-WP6-T2	2018	1 cm depth	ND*	8.03	6.81	**1.22**	ND*	15.83	13.22	**2.61**
		95% Conf		*+/- 0.46*	*+/- 0.41*	*+/- 0.18*		*+/- 0.72*	*+/- 0.67*	*+/- 0.18*

* Indicates site where only two temperatures were taken a day, at 6:00 AM and 6:00 PM. ΔT at these sites is therefore not precise and likely an underestimate of real value. Minimum temperatures are generally after 6 AM in April and Maximum temperatures 3-4 hours before 6 PM in June.

Table S3. Average chemical characteristics of soils used in this study. (NA- not available)

	Chemical variable (dry wt. basis)											
Soil	pH	Grav. Moisture (%)	OM (%)	P (ppm)	K (ppm)	Mg (ppm)	Ca (ppm)	NO_3^--N (ppm)	NH_4^+-N (ppm)	TKN (%)	CEC (meq/ 100 g)	Total Fe (%)
Havana (0-5 cm)	7.4	7.4	1.1	58.2	74.5	131.2	855.0	9.0	11.4	0.1	5.6	151.4
Havana (20-30 cm)	7.4	6.7	0.4	50.8	46.6	65.2	460.0	1.7	2.6	0.02	3.0	162.3
Urbana (0-5 cm)	6.1	20.7	4.1	43.6	198.4	384.4	2174.1	16.2	4.9	0.2	18.9	184.0
Lajas NEON site[a]	6.5	18.0	2.9	1255	7053	13,222	11,660	0	NA	0.3	NA	68

[a] Chemical data for soil from the Lajas site 0-30 cm is available at https://www.neonscience.org

T-RFLP Community Profiling Based on Amplification of 16S rRNA Genes.

The 16S rRNA genes in the DNA pools were amplified using primer set F27/1492R (Lane, 1991). The forward primer F27 (5'-AGA GTT TGA TCM TGG CTC AG-3') was labeled at the 5' end with 6-carboxyfluorescein (6-FAM) and when coupled to the reverse primer 1492 (5'-GGT TAC CTT GTT ACG ACT T-3'), amplifies the 16S rRNA genes from a broad diversity of bacteria. The primers were HPLC-purified and obtained from IDT (Integrated DNA Technologies, Skokie, IL, USA). Stock concentrations (100 µM) of each primer were made by adding Invitrogen™ UltraPure™ DNase/RNase-Free Distilled Water (Thermo Fisher Scientific Waltham, MA, USA) and subsequently diluted for use in PCR accordingly for amplification of the target gene. PCR reactions were performed in 50 µl volumes using the Takara ExTaq PCR kit (Clontech) and a MJ Research PTC-200 Gradient Thermal Cycler. The optimized reaction mixture was the following: 1X PCR buffer, 0.2 mM each deoxynucleoside triphosphate (dNTPs), 0.025U μL^{-1} TaKaRa Ex Taq DNA polymerase, 1.0 µM each forward and reverse primers, and ~1 ng DNA. T4 gene protein 32 (New England Biolabs, 10 mg/ml stock) was added to the reaction mixture to obtain a final concentration of 62.5 µg mL^{-1}. The following thermocycling program was used: initial denaturation step at 94°C for 5 min, followed by 25 cycles of 94 °C for 90 sec, 55 °C 90 sec, and 72 °C for 90 sec, then a final extension at 72 °C for 10 min. PCR products were resolved by gel electrophoresis in 2.5% High Resolution Agarose (fragments < 1 Kb) (Gold Biotechnology, Olivette, MO, USA) at 4 V cm^{-1} for 70 minutes or 1% Seakem LE Agarose (fragments > 1 Kb) (Lonza) at 4 V cm^{-1} for 50 minutes in 1X TBE buffer on a HU13 Midi horizontal gel unit (Scie-plas Ltd., Cambridge, UK). DNA ladders consisted of 10 µL of Quick-Load Purple 1kb DNA Ladder (0.1-10.0 kb) (New England Biolabs Inc., Ipswich, MA, USA) or 5 ul of 1kb DNA ladder (Gold Biotech). The PCR amplified mixture was purified further to remove excess primers and salts using the QIAquick PCR Purification Kit (Qiagen) following manufacturer's instructions except 30 ul of nucleic acids-free water was used to elute the DNA from the column and the final DNA suspension was stored at -20 °C until restriction enzyme digestion.

The amplified 16S rRNA gene products were digested using the restriction enzyme HaeIII, previously determined to be the most optimal for generating a wide range of terminal fragments (TRF) sizes generated following enzyme digestion of the amplicon pools typically resulting from local Illinois soils. Additional enzyme candidates for digestion include HhaI and HpaII and can be used instead of HaeIII. HaeIII digestion was performed for 12 h at 37 °C using the NEB restriction enzyme kit (New

England Biolabs) according to manufacturer's instructions. Digested fragments were visualized using 2.5% high resolution agarose gel electrophoresis along with reference DNA containing fragment sizes ≤ 1 kb (Quick-Load®Purple 1 kb Plus DNA Ladder). Terminal fragment size analysis was performed using services available at the Roy J. Carver Biotechnology Center, University of Illinois, Urbana, IL.

Microbial Community Analyses

The TRF fragments generated from each sample were analyzed in Genemapper v. 3.7 (Applied Biosystems), calibrated against size standards 50-1000 bp (ROX1000). Data was sorted according to size after removal of fragments <50 bp and >1000 bp fragments (outside the range of calibration), and a threshold <80 relative fluorescence intensity was determined as background fluorescence signals based on analysis of water samples used as blank controls. The resulting dataset were unique patterns (i.e. T-RFLP profiles) comprised of TRFs that represented the bacterial 16S rRNA gene pool amplified from DNA recovered from each sample. The technical replicates of one sample used for internal assessment of extraction and amplification variability yielded identical T-RFLP patterns.

To assess whether soil depth correspond to statistically different microbial communities as measured by 16S rRNA gene-based T-RFLP profiles, non-parametric permutational MANOVA (PERMANOVA) was used to analyze variances using the PERMANOVA+ package in PRIMER v.6 software (PRIMER-E Ltd, Plymouth, UK). To identify specific soil environmental variables that correlated to the patterns of microbial profiles, the non-parametric BIO-ENV algorithm available in PRIMER was used to test combinations of the variables which together result in resemblances among samples whose best rank order matches the rank order of the biological species resemblance. The correlation analysis was based on the community profile resemblance matrix calculated using Bray-Curtis similarity and Euclidian distance calculations for the environmental data. Comparisons of the Spearman coefficient (ρ) generated during BIO-ENV were used to assess an index (scale 0-1 representing the relative strength) corresponding to an environmental variable, or combination thereof, that correlated best with the community pattern found at the specified soil depths. While not explanatory, the analysis provides a parsimonious set of variables useful for downstream modeling methods (e.g. distance-based linear modeling).

Diurnal Temperature Microcosm Experiment

In June 2014, 0-5 cm and 25-30 cm depth bulk soil samples (~5 kg each) were collected from our field site in Havana, IL. This is a sandy soil, with chemical characteristics provided in Table S3. The soil samples were stored at 25 °C (representative of mean summer temperature) to minimize any extreme temperature disturbances to the microbial communities prior to use one week later in the microcosm experiment. The bulk soil sample from each depth was sieved sequentially through 4.75 mm and 1-2 mm sieves to remove rocks, plant detritus, and root material. The sieved and homogenized soil from each depth was split into two bags (700 g each per depth) corresponding to the two temperature treatments, diurnal T and constant T. The 24 h diurnal T regimen consisted of a continuous cycle of T_{min} = 18.9 °C and T_{max} = 37 °C (Δ18.1 °C) based on 0-5 cm temperature data

recorded in the field for a typical day in June (Fig 1B). The constant T regimen was held at 25 °C representing the midpoint between the average T at the 5 cm soil depth (25.5 °C) and at 25-30 cm soil depth (24.5 °C) for the same typical day in June. We felt it was important to use a single proxy temperature for the mean temperature at both depths to make comparative analyses easier.

To simulate the impact of added fertilizer that the Havana field site receives each year prior to planting, each bag of soil received ~240 µmoles total N (aq.) through addition of 12 ml of a urea ammonium-nitrate (UAN) N-fertilizer stock solution mixture prepared in site-collected irrigation water (stock solution: 10 mM urea, 20 mM NH_4Cl, 20 mM KNO_3). The addition of the N-fertilizer solution resulted in a final soil moisture content of 6.4% w/w, which is comparable to typical field moist conditions observed for that time of year. Following thorough mixing of the soil and N solution in each bag, the N-amended soil was split in 200 g aliquots into 473 mL jars for the microcosm experiment (n = 3 per temperature treatment and soil depth, total jars = 12). The remaining 100 g soil in each bag was used for time 0 baseline chemical analyses and was subsampled for initial microbial community characterization. The soil microcosms were incubated in the dark for 21 days in growth chamber incubators (Model E15, Controlled Environments, Inc., Pembina, ND) programmed for the diurnal temperature treatment or the constant temperature treatment. The jars were sealed with lids equipped with a thick butyl rubber stopper used as a gas sampling port. Gas tight conditions for each jar was checked prior to use by its ability to maintain 20% overpressure in the headspace. The jars with soil were weighed initially, and before and after any soil removal at sampling times to monitor for any loss in soil moisture, which was nominal throughout the duration of incubation. Gas sampling of the jar headspace for N_2O, CO_2 and O_2 analysis was performed at 0 h, 24 h, 48 h, 7 d, 10 d, 14 d, and 21 d. A 5 ml sample of the headspace gas was removed via the sampling port in the lid using a N_2-purged syringe and needle assembly equipped with a gas tight valve; the gas sample was transferred to and stored in a 8 ml N_2-purged gas tight vial sealed with a butyl rubber stopper. This created a vial with overpressure; effectively each 8 ml vial contained 15 ml of gas. The dilution effect was accounted for after analysis by GC. All gas samples taken during the course of the experiment were stored at 25 °C until analyzed together at the termination of incubation. Immediately after gas sampling, the jars were opened to remove soil subsamples for extraction of nucleic acids and analyses of NH_4^+, NO_3^-, NO_2^-, soil moisture and pH. This also insured that aerobic conditions were maintained in the headspace. The soil was mixed well before a ~5 g subsample was removed, added to 10 ml of 0.1 M KCl, placed on a shaker at room temperature for 1 h, and then centrifuged at 4 °C. The supernatant was filtered (0.45 µm) before transfer to a clean tube and stored at -20 °C prior to chemical analyses. Two 0.5 g soil subsamples were taken initially and at all subsequent time points from each replicate bottle, placed in 2 ml tubes with 1.0 ml of LifeGuard RNA preservative for future nucleic acid extraction. The tubes were mixed briefly and allowed to sit at room temperature for 24 h before being frozen at -20 °C until needed for further processing. For pH measurements soil subsamples (~2 g) were equilibrated at room temperature, mixed with Milli-Q deionized

water (2 mL), and analyzed using an electrode. At 0 h and 21 d soil moisture contents were also measured. Soil moisture was determined gravimetrically by drying ~5 g soil at 105 °C until constant mass was achieved.

RESULTS

Microbial Community Analyses—Indication that DTR is important.

Since the 0-5 cm core segment receives the largest DTR for many months of the year we evaluated whether this shallow community was significantly different from the 20-30 cm microbial community throughout seasons and years. At both Illinois agricultural field sites the microbial communities between all depth ranges were significantly different (significance level set at $p<0.05$) (Figure S3, Table S4), with seasonal and yearly differences also significant. In contrast to the well-drained sandy soil at Havana, there were larger intra-field (centroid) differences occurring at the Urbana site, where a natural slope yielded a legacy of drainage differences across the length of the field (Table S4). This larger intra-field difference, however, was more pronounced with the 20-30 cm depth than with the 0-5 cm depth. When comparing communities at the specified depths in pair-wise tests, the distinction between the 0-5 cm and 20-30 cm depths were the greatest at both locations (Table S4).

Our T-RFLP analysis of amplified 16S rRNA genes, used to capture community profiles that allow comparative assessment between samples, clearly show that the near surface microbial community is remarkably stable throughout the year at two climatologically similar agricultural field sites with contrasting soil types. This 0-5 cm community is significantly different from the community structure found from 20-30 cm, the latter characterized by a much smaller DTR (Figure S3). Remarkably, stability of the microbial community in the shallow depth was maintained following four years of crop rotation (soybeans and corn) and over all seasons. Even with no implicit assumptions about causation made about any specific environmental variable that included both temperature- and chemical-related factors, the single best factor was DTR, either alone or in combination with other environmental variables, that correlated with the patterns of community profiles in the surface and deep communities (Table S5).

RESULTS: Soil Microcosm Experiment- Laboratory observations of DTR on microbial activity

We compared cumulative carbon mineralization and N_2O flux from surface soil samples exposed to a large DTR and soil samples taken at 30 cm exposed to a small DTR. The UAN-amended shallow soil (0-5 cm) had higher cumulative N_2O and CO_2 generation when incubated with a diurnal temperature cycle (DTR=18.1 °C, Max T= 37 °C and mean T =25 °C) compared to incubations at the mean temperature (25 °C) (Figure S4A and S4B). In contrast, the soil microcosms containing the deeper soil (25-30 cm) conducted correspondingly with the same two temperature regimens demonstrated no difference in cumulative N_2O and CO_2 generation. Results indicated that shallow soils exhibited a differential response to fertilizer when a large DTR was implemented compared to constant temperature exposure. This was in contrast with the response to nitrogen fertilizer responses in deeper soils where large DTRs do not naturally occur.

Our experimental results support the hypothesis that DTR presents a selective force for biochemical adaptation in shallow soil microbial communities. This adaptive response appears to be muted in deeper soils where the DTR is relatively small. The adaptive characteristics could be associated with individual populations (genetic changes – circadian clocks) or with the community as a whole (temporal mutualism). Our experimental results and the list of studies presented in Table 2 provide data that are consistent with the conceptual belief that established communities are temperature adapted to conditions found in their natural habitat. While we do not consider our experiments to provide the definitive proof for temperature adaptation, in combination with previous soil related studies and evidence for genetically triggered responses to DTR the case becomes very strong that such adaptation has occurred over life's history on Earth.

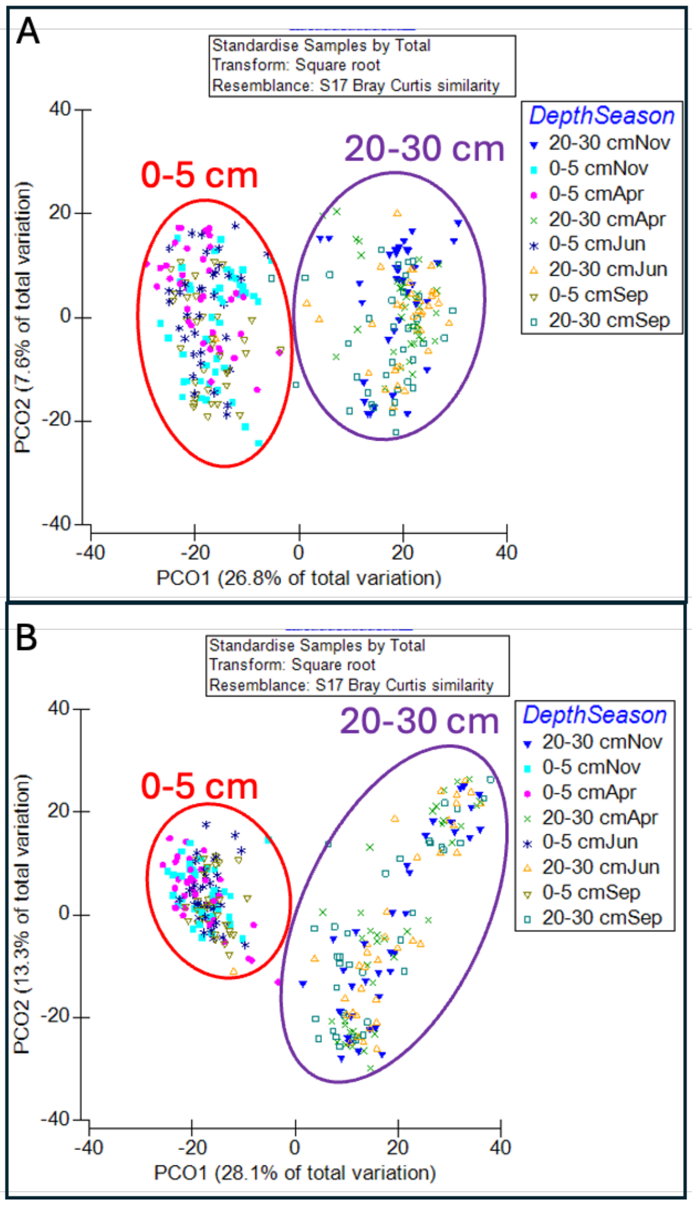

Figure S3. PCO ordination of the bacterial communities based on T-RFLP of 16S rRNA gene amplicons using Bray-Curtis dissimilarities as a measure of the beta-diversity of samples taken from 0-5 cm and 20-30 cm from 2011-2015 at four times a year. Panels A and B show the community analysis results from agricultural field sites in Havana, IL and Urbana, IL, respectively. Bacterial communities near the surface where a significant DTR occurs are significantly different from communities found at depth where the DTR is small.

Table S4. Results of permutation ANOVA (PERMANOVA) of Bray-Curtis dissimilarities calculated between microbial community assemblages based on 16S rDNA T-RFLP profiling of samples from soil depths at Havana and Urbana field sites from Nov. 2011 to Sep. 2015. Significant differences are shown at p-level ≤0.05 between samples according to factors of soil depth, season, year, and intrafield location (centroid).

Factor	*df*	*SS*	*MS*	*Pseudo-F*	*P(perm)*	*Unique Perms*
Havana:						
**Depth*	4	1.4164E5	35411	42.482	**0.001**	9809
***Season*	3	12125	4041.8	4.8489	**0.001**	9825
Year	4	40679	10170	12.201	**0.001**	9765
****Centroid*	2	10666	5333	6.3979	**0.001**	9844
Residuals	539	4.4928E5	833.55			
Total	556	7.0592E5				
Urbana:						
**Depth*	4	1.3946E5	34866	50.75	**0.001**	9843
***Season*	3	9323.4	3107.8	4.5237	**0.001**	9829
Year	4	27166	6791.4	9.8855	**0.001**	9803
****Centroid*	2	70506	35253	51.314	**0.001**	9879
Residuals	539	3.703E5	687.01			
Total	556	6.5168E5				

Abbreviations: *df*, degree of freedom; *SS*, sum of squares; *MS*, mean square error.
*0-5 cm, 5-10 cm (Nov. 2011 only), 10-20 cm (Nov. 2011 only), 5-20 cm, 20-30 cm soil depths.
** April, June, September, and November seasonal sampling times.
***Three intra-field fixed locations used for soil sampling.

Table S5. Combinations of environmental variables[a] resulting in the best match (Spearman correlation coefficients, ρ) of the rank order of resemblances among samples to those of the communities analyzed using T-RFLP profiling based on amplification of the 16S rRNA genes. Note DTR (average diurnal temperature range) is included in every combination of chemical and temperature variables showing the highest correlations. The most positively correlated environmental variables found in combination with DTR include pH, total Fe, TKN, OM, nitrate-N (NO_3^-—N), MaxT and MinT.

Location	Soil chemical and temperature variables[a]<br>ρ[b]						
**Urbana, IL Site**	DTR	Fe<br>DTR	pH<br>Fe<br>DTR	TKN<br>pH<br>Fe<br>DTR	TKN<br>Fe<br>DTR	OM<br>pH<br>Fe<br>DTR	Max T<br>pH<br>Fe<br>DTR
0-5cm, 20-30cm		0.581	0.590	0.585	0.575		0.566
0-5cm, 5-20cm, 20-30cm	0.458	0.464	0.469			0.442	0.437
**Havana, IL Site**	DTR	OM<br>DTR	Min T<br>OM<br>DTR	OM<br>Fe<br>DTR	OM<br>NO_3^--N<br>DTR	TKN<br>Min T<br>OM<br>DTR	Max T<br>OM<br>DTR
0-5cm, 20-30cm	0.595	0.572	0.568		0.565	0.565	0.562
0-5cm, 5-20cm, 20-30cm	0.484	0.465	0.448	0.447	0.443		0.434

[a]Non-parametric method (BEST-ENV algorithm) used for calculation of correlation values (ρ) using 11 environmental variables including: pH, soil gravimetric moisture content, organic matter (OM), NO^{3-}-N, NH_4^+-N, total Kjeldahl N (TKN), total Fe (Fe), diurnal T range (DTR), mean maximum soil T at indicated depth (MaxT), mean minimum soil T at indicated depth (MinT), and mean T at indicated depth.
[b] Spearman correlation coefficient ρ> 0.5 → indicate moderate positive correlation. Coefficients ρ> 0.3 →indicate low positive correlation.

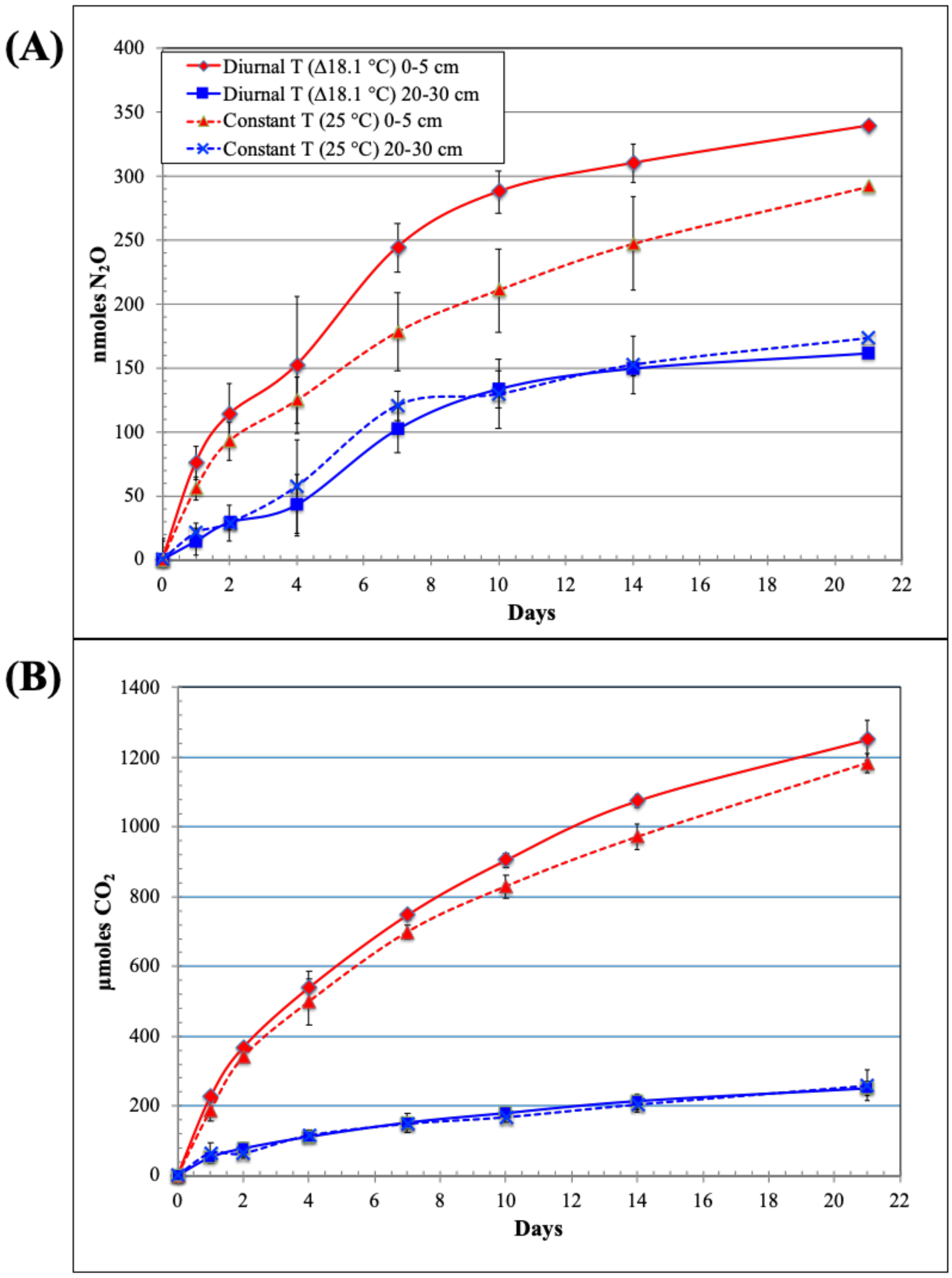

Figure S4. Cumulative **(A)** N_2O and **(B)** CO_2 generation in Experiment 1 microcosms containing shallow surface soil (Havana 0-5 cm) or deeper soil (Havana 20-30 cm) incubated under different temperature regimens: Diurnal - 18.9 °C - 37 °C (Δ18.1 °C), Constant Temp. – 25 °C , typical of June at the surface in the Havana field location (see Figure 1B). Error bars indicate the standard deviation based on triplicate measurements. Only shallow soils incubated under diurnal temperature conditions yielded a higher N_2O and CO_2 generation.

REFERENCES

Jackson, C., Harper, J., Willoughby, D., Roden, E. & Churchill, P. (1997) A simple, efficient method for the separation of humic substances and DNA from environmental samples. *Applied and Environmental Microbiology*, 63(12), 4993-4995.

Lane, D. J. (1991) 16S/23S rRNA sequencing., in Stackebrandt, E. & Goodfellow, M. (eds), *Nucleic acid techniques in bacterial systematics*. New York: John Wiley and Sons, 115-175.

Orellana, L. H., Chee-Sanford, J. C., Sanford, R. A., Löffler, F. E. & Konstantinidis, K. T. (2018) Year-round shotgun metagenomes reveal stable microbial communities in agricultural soils and novel ammonia oxidizers responding to fertilization. *Applied and Environmental Microbiology*, 84(2), e01646-17.

Orellana, L. H., Hatt, J. K., Iyer, R., Chourey, K., Hettich, R. L., Spain, J. C., Yang, W. H., Chee-Sanford, J. C., Sanford, R. A., Löffler, F. E. & Konstantinidis, K. T. (2019) Comparing DNA, RNA and protein levels for measuring microbial dynamics in soil microcosms amended with nitrogen fertilizer. *Scientific Reports*, 9(1), 17630.

Tsai, Y. & Olson, B. (1991) Rapid method for direct extraction of DNA from soil and sediments. *Applied and Environmental Microbiology*, 57(4), 1070-1074.
